# Corticosteroid suppression of antiviral immunity increases bacterial loads and mucus production in COPD exacerbations

**DOI:** 10.1038/s41467-018-04574-1

**Published:** 2018-06-08

**Authors:** Aran Singanayagam, Nicholas Glanville, Jason L. Girkin, Yee Man Ching, Andrea Marcellini, James D. Porter, Marie Toussaint, Ross P. Walton, Lydia J. Finney, Julia Aniscenko, Jie Zhu, Maria-Belen Trujillo-Torralbo, Maria Adelaide Calderazzo, Chris Grainge, Su-Ling Loo, Punnam Chander Veerati, Prabuddha S. Pathinayake, Kristy S. Nichol, Andrew T. Reid, Phillip L. James, Roberto Solari, Peter A. B. Wark, Darryl A. Knight, Miriam F. Moffatt, William O. Cookson, Michael R. Edwards, Patrick Mallia, Nathan W. Bartlett, Sebastian L. Johnston

**Affiliations:** 1 COPD and Asthma Section, National Heart and Lung Institute, Imperial College London, Norfolk Place, London, W2 1PG UK; 2 Faculty of Health and Medicine and Priority Research Centre for Healthy Lungs, Hunter Medical Research Institute and University of Newcastle, Newcastle, NSW 2305 Australia; 3 Genomic Medicine, National Heart and Lung Institute, Imperial College London, Cale Street, London, SW3 6LY UK

## Abstract

Inhaled corticosteroids (ICS) have limited efficacy in reducing chronic obstructive pulmonary disease (COPD) exacerbations and increase pneumonia risk, through unknown mechanisms. Rhinoviruses precipitate most exacerbations and increase susceptibility to secondary bacterial infections. Here, we show that the ICS fluticasone propionate (FP) impairs innate and acquired antiviral immune responses leading to delayed virus clearance and previously unrecognised adverse effects of enhanced mucus, impaired antimicrobial peptide secretion and increased pulmonary bacterial load during virus-induced exacerbations. Exogenous interferon-β reverses these effects. FP suppression of interferon may occur through inhibition of TLR3- and RIG-I virus-sensing pathways. Mice deficient in the type I interferon-α/β receptor (*IFNAR1*^−/−^) have suppressed antimicrobial peptide and enhanced mucin responses to rhinovirus infection. This study identifies type I interferon as a central regulator of antibacterial immunity and mucus production. Suppression of interferon by ICS during virus-induced COPD exacerbations likely mediates pneumonia risk and raises suggestion that inhaled interferon-β therapy may protect.

## Abstract (web summary)

Corticosteroid therapy is frequently used for chronic obstructive pulmonary disease (COPD) but its use is associated with increased risk of pneumonia. Here the authors show that corticosteroid use impairs innate and adaptive immunity to rhinovirus infection, which is restored by exogenous IFNβ.

## Introduction

Acute exacerbations of airway diseases are a major cause of morbidity and mortality. Rhinoviruses (RVs) are the most frequent cause of exacerbations, and experimental challenge studies in asthma and chronic obstructive pulmonary disease (COPD) confirm a causal role between RV infection and exacerbation^1–3^. The innate immune response to RV involves production of type I and III interferons (IFNs) by airway epithelial and immune cells. IFN responses to virus infection are impaired in asthma^4–7^ and COPD^1, 8^ and are related to increased exacerbation severity^6^.

Inhaled corticosteroids (ICS) are frequently prescribed therapies in airway diseases but only modestly reduce COPD exacerbation frequency^9–11^. Clinical trials report increased pneumonia risk with ICS use in COPD^9,12,13^, although the mechanisms underlying this effect are unknown. More severe RV infection increases frequency of secondary bacterial infections in COPD^14^, suggesting that the increased pneumonia risk may be a consequence of increased severity of initial virus infection.

Emerging evidence suggests that corticosteroids may impair innate antiviral immune responses^15,16^, but no studies have assessed the effects of ICS on host antiviral and antibacterial responses specifically in COPD, a disease associated with insensitivity to corticosteroids^17,18^. The precise mechanisms, including the role of type I IFN, underlying adverse effects have not been elucidated.

Here, using models of rhinovirus infection and rhinovirus-induced COPD exacerbation, we demonstrate that the commonly used ICS fluticasone propionate (FP) impairs innate and acquired antiviral immune responses leading to delayed virus clearance, mucus hypersecretion and increased lung bacterial loads. Our studies highlight a central role for type I IFN in regulating antimicrobial immunity and mucus secretion during virus-induced exacerbations. The adverse effects associated with immune suppression by ICS provide a mechanism to explain the increased risk of pneumonia associated with their use in COPD.

## Results

### FP affects transcription factors regulating immunity to RV

**Fig. 1:**
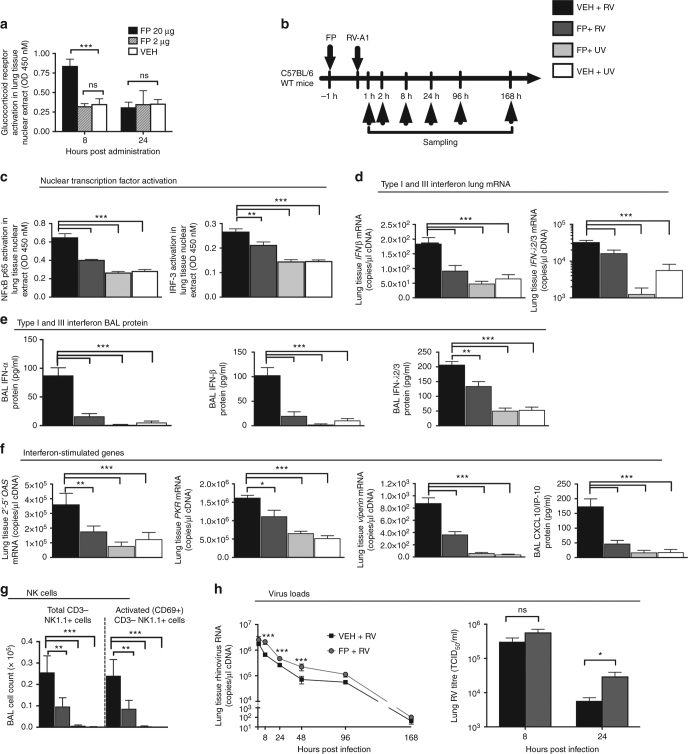
Fluticasone propionate suppresses innate antiviral immune responses and delays virus clearance in mice. **a** C57BL/6 mice were treated intranasally with fluticasone propionate (20 or 2 μg) or vehicle DMSO control. Glucocorticoid receptor activation in lung tissue was assessed by measuring nuclear DNA binding by ELISA. **b** C57BL/6 mice were treated intranasally with fluticasone propionate (20 μg) or vehicle DMSO control and challenged intranasally with rhinovirus (RV)-A1 or UV-inactivated RV-A1 (UV). **c** NFkB P65 subunit (8 h) and IRF-3 (2 h) activation in lung tissue was assessed by measuring nuclear DNA binding by ELISA. **d** *IFNβ* and *IFNλ2/3* mRNAs in lung tissue at 8 h post infection were measured by quantitative PCR. **e** IFN-α, IFN-β and IFN-λ2/3 proteins in bronchoalveolar lavage (BAL) at 24 h post infection were measured by ELISA. **f** Interferon-stimulated genes *2′–5′* *OAS*, *PKR* and *Viperin* mRNAs in lung tissue at 8 h post infection were measured by quantitative PCR. **g** BAL cells were stained for CD3 and the NK cell marker NK1.1 and additionally for the early activation marker CD69 and analysed by flow cytometry. BAL CD3^−^ NK1.1^+^ cell numbers and BAL CD3^−^ NK1.1^+^ CD69^+^ cell numbers were enumerated at day 2 post infection. **h** Rhinovirus RNA copies in lung tissue were measured by Taqman quantitative PCR and infectious virus in lung tissue was measured by titration in HeLa cells. Data represents mean (±SEM) of five–eight mice per treatment group, representative of at least two independent experiments. Data were analysed by one- or two-way ANOVA with Bonferroni post test. ns non-significant. *p < 0.05, **p < 0.01, ***p < 0.001

To determine the efficacy and duration of action of ICS in a mouse model of FP treatment, we assessed glucocorticoid receptor (GR) activation by measuring nuclear GR-DNA binding. Administration of 20 μg FP, a dose similar to that employed in previous animal studies^16,19^, significantly increased GR activation, while a tenfold lower dose did not (Fig. 1a). RV infection activates immune-regulating transcription factors, including nuclear factor kappa-B (NFκB), required for proinflammatory responses to RV infection^20^ and IFN regulatory factor-3 (IRF-3), required for antiviral responses^21^. We assessed the effect of FP administration (Fig. 1b) on RV activation of these transcription factors in mouse lung. FP suppressed lung NFκB p65 activation in RV-infected mice at 8 h post infection (Fig. 1c). For IRF-3, activation by RV occurred at 2 h post infection and significant suppression was also observed in FP-treated mice (Fig. 1c).

### ICS suppress innate immunity and impair virus control

We next assessed the effect of FP on innate antiviral immune responses to RV infection in mice. FP suppressed RV induction of lung *IFNβ* and *IFNλ2/3* mRNAs (~75% and ~60% reductions, respectively, Fig. 1d) and IFN-α, IFN-β and IFN-λ2/3 BAL proteins (~85%, ~80% and ~50% reductions, respectively, Fig. 1e). FP also suppressed RV induction of the interferon-stimulated genes (ISGs) 2′–5′*-oligoadenylate synthetase* (*2*′*–5*′*-OAS*), *protein kinase-R* (*PKR*) and *viperin* and CXCL10/IP-10 protein in BAL (~75%, ~45%, ~60% and ~80% reductions, respectively, Fig. 1f).

Type I IFN receptor signalling is required for airway natural killer (NK) cell responses to RVs^20^. Reduced numbers of total and activated (CD69 expressing) NK cells were observed in RV-infected mice treated with FP (Fig. 1g). Consistent with the suppression of innate antiviral responses (Fig. 1d–g), FP treatment led to delayed virus clearance with increased RV RNA copies in FP-treated mice at 8, 24 and 48 h post infection (maximal effect size threefold at 8 and 48 h) and increased levels of infectious virus at 24 h (Fig. 1h).

To confirm that other ICS compounds apart from FP can also impair antiviral immunity, we evaluated the effect of another commonly used agent, budesonide, on RV infection in mice. Administration of 20 μg of budesonide similarly suppressed RV induction of lung *IFNβ* and *IFNλ2/3* and increased RV RNA (Supplementary Fig 1a–d).

### FP impairs adaptive immune responses to RV infection

**Fig. 2:**
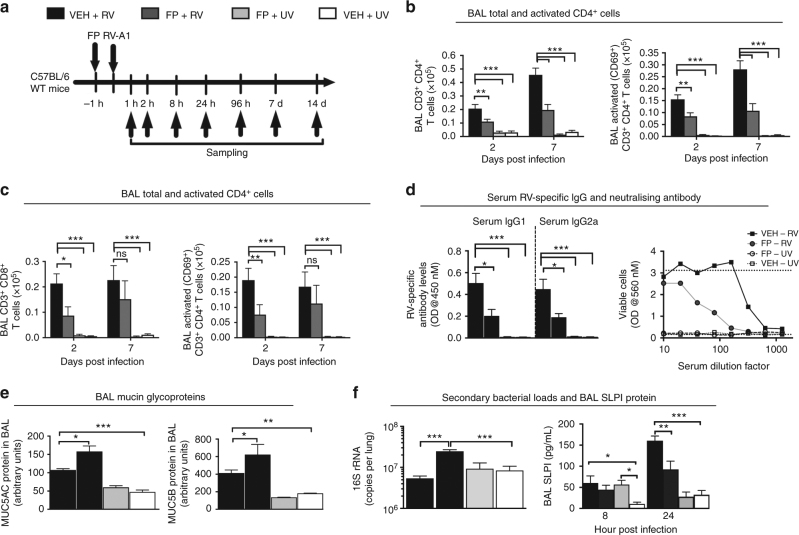
Fluticasone propionate suppresses acquired responses and augments mucin production and bacterial loads in mice. **a** C57BL/6 mice were treated intranasally with fluticasone propionate (20 μg) or vehicle DMSO control and challenged intranasally with rhinovirus (RV)-A1 or UV-inactivated RV-A1. **b**, **c** BAL cells were stained with antibodies specific for CD3, CD4, CD8 and CD69 and analysed by flow cytometry. **b** BAL CD3^+^ CD4^+^ T cell numbers and BAL CD3^+^ CD4^+^ CD69^+^ T cell numbers at day 7 post infection. **c** BAL CD3^+^ CD8^+^ T cell numbers and BAL CD3^+^ CD8^+^ CD69^+^ T cell numbers at day 2 post infection were evaluated. **d** Peripheral blood was harvested at day 14 post infection. RV-specific IgG1 and RV-specific IgG2A were quantified in serum by ELISA. Sera were assayed for their ability to prevent cytopathic effect caused by the same RV serotype used for in vivo challenge. Cytopathic effect was quantified by crystal violet staining. Top dotted line: serum only, (uninfected) controls. Bottom dotted line: virus infected (no serum) control. **e** MUC5AC and MUC5B proteins in BAL at day 7 post infection were measured by ELISA. **f** Bacterial copy number of 16S rRNA in lung tissue at 96 h was measured by quantitative PCR. Secretory leucocyte protease inhibitor (SLPI) protein in BAL was measured by ELISA. In all figures except **d** (right), data represents mean (±SEM) of five–eight mice per treatment group, representative of at least two independent experiments. Data were analysed by one- or two-way ANOVA with Bonferroni post test. ns non-significant; *p < 0.05, **p < 0.01, ***p < 0.001. In **d** (right), data points represent sera pooled from five mice per group, representative of two independent experiments

We next determined the effects of FP on adaptive immune responses to RV infection (Fig. 2a). Total and activated numbers of both CD4^+^ and CD8^+^ T cells were suppressed in FP-treated mice (Fig. 2b, c) as were serum levels of RV-specific IgG1 and IgG2a antibodies and neutralising antibody (Fig. 2d).

### FP suppresses virus-induced airway inflammation

FP administration prior to RV infection (Supplementary Fig. 2a) suppressed cellular airway inflammation with reduced numbers of neutrophils, lymphocytes and macrophages observed in BAL (Supplementary Fig. 2b). FP also suppressed RV induction of BAL neutrophil chemokines CXCL1/KC and CXCL2/MIP-2 and the lymphocyte chemokine CCL5/RANTES (Supplementary Fig. 2c).

### FP enhances mucin production in RV-infected mice

We evaluated the effect of FP on BAL fluid concentrations of the mucins MUC5AC and MUC5B, the major glycoprotein constituents of airway mucus^22^. RV infection alone increased airway production of both mucin glycoproteins and both were significantly further increased by FP treatment (~85% for MUC5AC and ~80% for MUC5B, Fig. 2e).

### FP impairs antibacterial responses in RV-infected mice

To further evaluate adverse effects associated with FP administration during RV infection, we assessed lung bacterial loads by measurement of bacterial 16S rRNA. FP increased bacterial loads (~2.5-fold) in RV-infected mice (Fig. 2f). FP also suppressed RV induction of secretory leukoprotease inhibitor (SLPI) protein (~50% reduction, Fig. 2f), an antimicrobial peptide (AMP) implicated in protection against secondary bacterial infection following RV infection in COPD^14^. FP administration also suppressed RV induction of proinflammatory cytokines IL-6 and TNF (Supplementary Fig. 2d) and the AMP pentraxin-3, but had no effect on RV induction of other AMPs α-defensin 1, β-defensin 2 or mannose-binding lectin-2 (Supplementary Fig. 2e). The AMPs surfactant protein-D and lysozyme were not significantly induced by RV infection nor suppressed by FP in mice (Supplementary Fig. 2f).

### FP suppresses TLR3- and RIG-I-mediated IFN responses

**Fig. 3:**
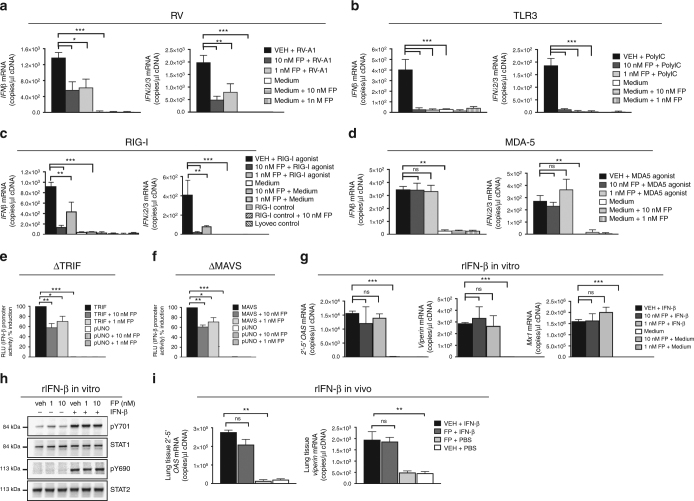
Fluticasone propionate suppresses TLR3 and RIG-I-induced interferon responses but does not block type I IFN signalling. **a**–**d** BEAS-2B cells were treated with fluticasone propionate at 1 and 10 nM concentrations and stimulated with rhinovirus-A1 or receptor-specific agonists. Cell lysates were collected at 24 h post stimulation. **a** *IFNβ* and *IFNλ2/3* mRNA expression following RV-A1 stimulation was measured by quantitative PCR. **b** *IFNβ* and *IFNλ2/3* mRNA expression following poly(I:C) stimulation was measured by quantitative PCR. **c** *IFNβ* and *IFNλ2/3* mRNA expression following RIG-I agonist (transfected 5′ppp-dsRNA) stimulation was measured by quantitative PCR. Transfected dsRNA lacking the 5′ triphosphate was used as a ‘RIG-I control’. **d** *IFNβ* and *IFNλ2/3* mRNA expression following MDA-5 agonist (transfected HMW Poly(I:C) directly pre-coupled to the transfection reagent LyoVec) stimulation was measured by quantitative PCR. LyoVec without Poly(I:C) (LyoVec control) was used as a control for transfection. **e**, **f** BEAS-2B cells were transfected with interferon-β promoter–reporter constructs together with ΔTRIF, ΔMAVS or vector control (pUNO1) and then treated with fluticasone propionate (FP) 1 or 10 nM or medium control 3 h later. Cells were collected 24 h later and relative light units (RLU) were determined. **e** *IFNβ* promoter activity following transfection with ΔTRIF. **f** *IFNβ* promoter activity following transfection with ΔMAVS. **g**, **h** BEAS-2B cells were treated with fluticasone propionate at 1 and 10 nM concentrations and stimulated with recombinant IFN-β. **g** *2*′–*5*′ *OAS, viperin* and *MX1* mRNA expression at 24 h post stimulation was measured by quantitative PCR. **h** Cell extracts were collected at 1 h post stimulation and analysed by western blotting with antibodies to pSTAT1 Y701, STAT1, pSTAT2 Y690 and STAT2. In **a**–**g**, data represent mean (±SEM) comprising three independent experiments, analysed by one-way ANOVA with Bonferroni post test. ns non-significant. *p < 0.05, **p < 0.01, ***p < 0.001. In **h**, data shown are representative of results from three independent experiments. **i** C57BL/6 mice were treated intranasally with fluticasone propionate (20 μg) or vehicle DMSO control and additionally with recombinant 10^4^ units IFN-β. Interferon-stimulated genes *2*′-*5*′ *OAS*, and *Viperin* mRNAs in lung tissue at 8 h post infection were measured by quantitative PCR. Data represents mean (±SEM) of five mice per treatment group, representative of at least two independent experiments. Data were analysed by one-way ANOVA with Bonferroni post test. ns non-significant. *p < 0.05, **p < 0.01, ***p < 0.001

To investigate mechanisms whereby FP suppresses innate and acquired immune responses, we next assessed which pattern recognition receptor (PRR) and IFN signalling pathways were inhibited by FP in human BEAS-2B bronchial epithelial cells. As observed in mice in vivo, FP treatment of BEAS-2B cells in vitro suppressed RV induction of *IFNβ* and *IFNλ2/3* (Fig. 3a). Double-stranded RNA intermediates form during RV replication and activate the PRRs TLR3, RIG-I and MDA-5^21,23^. FP treatment markedly suppressed induction of *IFNβ* and *IFNλ2/3* by TLR3 (Fig. 3b) and RIG-I agonists (Fig. 3c) but had no effect on MDA-5 agonist (Fig. 3d).

TLR3 induces IFN expression via the adaptor molecule TIR domain-containing adaptor inducing IFN-β (TRIF), while MDA-5 and RIG-I signal via mitochondrial antiviral signalling protein (MAVS)^23,24^. We therefore transfected BEAS-2B cells with *IFNβ* promoter–reporter constructs and stimulated using plasmids encoding constitutively active TRIF (ΔTRIF) or MAVS (ΔMAVS). Transfection with ΔTRIF or ΔMAVS significantly increased *IFNβ* promoter activity (Fig. 3e, f). Consistent with our finding that FP suppressed TLR3- and RIG-I-mediated IFN induction, treatment with FP suppressed both ΔTRIF- and ΔMAVS-induced *IFNβ* promoter activity (Fig. 3e, f).

Secondary amplification of virus-induced IFN release involves type I IFN acting in an autocrine manner via the IFN alpha/beta receptor (IFNAR) to stimulate further induction of IFNs and ISGs^20^. To assess effects of FP on IFN signalling, we stimulated BEAS-2B cells with recombinant IFN-β and found no effect of FP on IFN-β induction of ISGs *2*′*–5*′*-OAS*, *viperin* and *myxovirus resistance A* (*Mx1*) (Fig. 3g), thereby demonstrating that FP suppression of IFN is restricted to effects on initial virus-sensing pathways and not secondary amplification via IFNAR. IFN signalling via IFNAR induces phosphorylation of STAT1/2. Therefore, to further confirm this finding, immunodetection of STAT1 phosphorylated at Y701 (pY701) and STAT2 phosphorylated at Y690 (pY690) was investigated in BEAS-2B cells stimulated with recombinant IFN-β. FP had no effect on IFN-β induction of either STAT1 or STAT2 phosphorylation (Fig. 3h). We additionally confirmed these in vitro findings by demonstrating that FP administration had no effect on lung ISG induction following recombinant IFN-β administration in mice (Fig. 3i).

### IFNβ reduces virus load and reverses mucin augmentation by FP

**Fig. 4:**
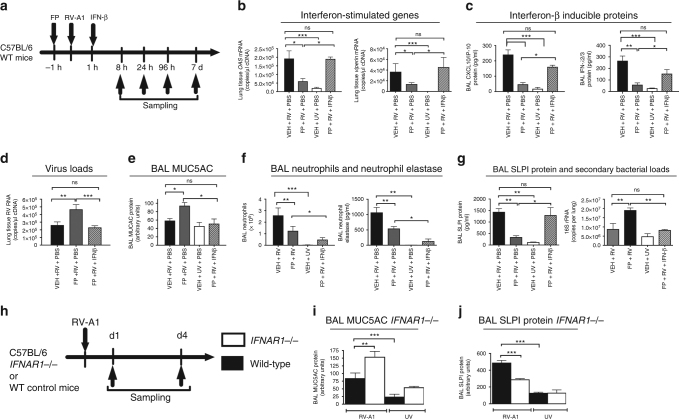
Type I IFN augments antimicrobial responses and suppresses MUC5AC and bacterial loads. **a** C57BL/6 mice were treated intranasally with fluticasone propionate (20 μg) or vehicle DMSO control and challenged intranasally with rhinovirus (RV)-A1 or UV-inactivated RV-A1 (UV). One hour after RV challenge, mice were additionally treated intranasally with 10^4^ units recombinant IFN-β or PBS control. **b** *2′–5′* *OAS* and *viperin* mRNAs in lung tissue were measured by quantitative PCR at 8 h post infection. **c** CXCL10/IP-10 and IFN-λ2/3 proteins were measured in bronchoalveolar lavage (BAL) by ELISA at 24 h post infection. **d** RV RNA copies in lung tissue were measured by quantitative PCR at 24 h post infection. **e** MUC5AC protein in BAL was measured by ELISA at day 7 post infection. **f** BAL neutrophils were enumerated by cytospin assay and neutrophil elastase protein was measured in BAL by ELISA at 8 h post infection. **g** Secretory leucocyte protease inhibitor (SLPI) protein in bronchoalveolar lavage (BAL) at 24 h post infection was measured by ELISA. 16S rRNA copies in lung tissue were measured by quantitative PCR at 96 h post infection. **h** Wild-type or *IFNAR1*^*−/−*^ C57BL/6 mice were challenged intranasally with rhinovirus (RV)-A1 or UV-inactivated RV-A1 (UV). **i** MUC5AC protein at day 4 and **j** secretory leucocyte protease inhibitor (SLPI) protein at day 1 post infection was measured in BAL by ELISA. Data represents mean (±SEM) of five–eight mice per treatment group, representative of at least two independent experiments. Data were analysed by one-way ANOVA with Bonferroni post test. ns non-significant. *p < 0.05, **p < 0.01, ***p < 0.001

To demonstrate that suppression of IFN by FP is functionally related to increased virus load and mucin production, we administered recombinant IFN-β in combination with FP treatment and RV infection (Fig. 4a). For clarity, selected experimental groups are in Fig. 4 and the full data set in Supplementary Fig. 3. IFN-β reversed FP-mediated suppression of lung *2*′*–5*′*-OAS* and *viperin* expression (Fig. 4b) and of BAL CXCL10/IP-10 and IFN-λ2/3 protein concentrations (Fig. 4c). Compared to FP-treated RV-infected mice, administration of IFN-β following FP treatment and infection significantly reduced virus loads to levels observed in untreated virus-infected mice (Fig. 4d). IFN-β treatment additionally prevented FP upregulation of BAL MUC5AC glycoprotein levels (Fig. 4e).

### IFNβ reverses FP impairment of antibacterial immunity

Secondary bacterial infection in RV-induced COPD exacerbations is mediated by virus-induced neutrophil elastase-mediated degradation of AMPs, including SLPI^14^. We therefore investigated whether IFN-β could prevent this occurring. IFN-β treatment further enhanced FP suppression of BAL neutrophil numbers and neutrophil elastase levels (Fig. 4f), reversed FP suppression of BAL SLPI levels and prevented FP augmentation of bacterial load (Fig. 4g) in RV-infected mice. However, IFN-β treatment had no effect on FP suppression of antibacterial mediators IL-6, TNF or pentraxin-3 (Supplementary Fig. 4a–c).

### *IFNAR1*^−/−^ mice have altered mucin and SLPI responses to RV

To confirm suppression of IFN is functionally related to enhanced mucin and impaired antibacterial immunity, we evaluated MUC5AC and SLPI expression in RV-infected mice deficient in IFNAR signalling (*IFNAR1*^−/−^) (Fig. 4h). BAL MUC5AC protein concentrations in RV-infected *IFNAR1*^*−/−*^ mice were increased (Fig. 4i). To confirm that IFNAR signalling is also important for SLPI production during RV infection, we measured BAL SLPI protein in RV-infected *IFNAR1*^*−/−*^ mice and observed reduced concentrations (Fig. 4j).

### IFNβ does not restore acquired responses nor reduce MUC5B

We observed no effect of IFN-β treatment on FP suppression of adaptive immune responses with no difference in BAL CD4^+^ or CD8^+^ T cell numbers or serum-neutralising antibody levels in RV-infected mice treated with FP and IFN-β versus FP alone (Supplementary Fig. 4d). In contrast to the effect observed on MUC5AC (Fig. 4e), recombinant IFN-β also had no effect on FP enhancement of BAL MUC5B protein levels (Supplementary Fig. 4e).

### FP suppresses IFN and enhances mucus secretion in COPD cells

**Fig. 5:**
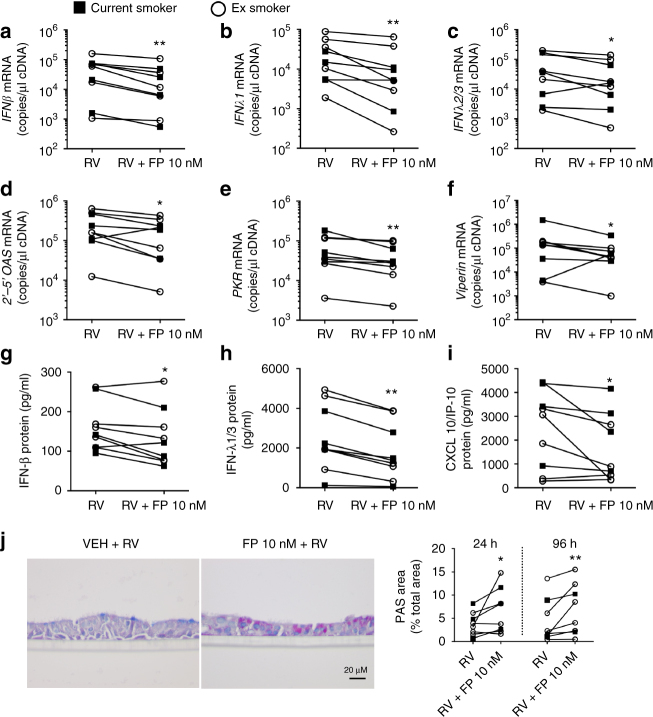
Fluticasone propionate impairs ex vivo antiviral immune responses and enhances mucus secretion in COPD cells. Primary airway epithelial cells (AECs) from nine patients with GOLD stage III COPD were cultured at the air–liquid interface and then treated in vitro with fluticasone propionate at 1 and 10 nM concentrations or vehicle control and infected with rhinovirus (RV)-A1. Cell lysates and supernatants were collected post infection. **a** *IFNβ*, **b** *IFNλ1*, **c** *IFNλ2/3*, **d** *2*′*–5*′ *OAS*, **e** *PKR* and **f** *viperin* mRNA expression in cell lysates at 48 h was measured by quantitative PCR. **g** IFN-β, **h** IFN-λ1/3 proteins at 96 h and **i** CXCL10/IP-10 protein at 72 h in cell supernatants were measured by ELISA. **j** AECs for eight patients were paraffin-embedded and stained with periodic acid-Schiff (PAS). Left: Representative images of cells treated with vehicle + RV and FP10 nM + RV are shown. Scale bars: 20 μm, Magnification, x400. Right: Scoring for PAS-positive mucus-producing cells at 24 and 96 h post infection. Data represents individual patients and analysed by Mann–Whitney *U*-test. *p < 0.05, **p < 0.01, ***p < 0.001

We next investigated whether similar adverse effects of FP occurred in cells from COPD patients. We evaluated the effects of FP in airway epithelial cells (AECs) sampled bronchoscopically from nine patients with GOLD stage 3 disease, cultured at an air–liquid interface. The quality of cultures was confirmed by the presence of ciliated epithelium, mucus-producing cells, pseudostratified structure and assessment of trans-epithelial electrical resistance (Supplementary Fig. 5a). Demographic/clinical characteristics of the patients are in Table 1. FP significantly suppressed early RV induction of *IFNβ* and *IFNλ1* and *IFNλ2/3* (Fig. 5a–c and Supplementary Fig. 6a). FP also suppressed RV induction of *2*′*–5*′-*OAS*, *PKR* and *viperin* (Fig. 5d–f) and additionally suppressed IFN-β, IFN-λ1/3 and CXCL10/IP-10 proteins in supernatants of COPD AECs (Fig. 5g–i).

**Table 1:** Baseline characteristics of patients with COPD undergoing bronchoscopic sampling for airway epithelial cell experiments shown in Fig. 5

Subject	Age	Gender	GOLD stage	Smoking history	ICS use
1	87	Male	3	Ex-smoker	Fluticasone/Salmeterol
2	77	Male	3	Ex-smoker	Fluticasone/Salmeterol
3	77	Male	3	Ex-smoker	Fluticasone/Salmeterol
4	60	Male	3	Current	Nil
5	68	Female	3	Current	Fluticasone/Salmeterol
6	69	Male	3	Current	Nil
7	72	Male	3	Ex-smoker	Nil
8	59	Female	3	Ex-smoker	Fluticasone/Salmeterol
9	65	Female	3	Current	Nil

Each row represents an individual subject

In keeping with our finding that FP administration augmented mucin production in response to RV infection in mice, we also observed an increase in mucus staining associated with FP treatment in RV-infected human COPD AECs in *n* = 8 subjects for whom sample was available for histological analysis (Fig. 5j).

### FP impairs IFN responses in a COPD exacerbation mouse model

**Fig. 6:**
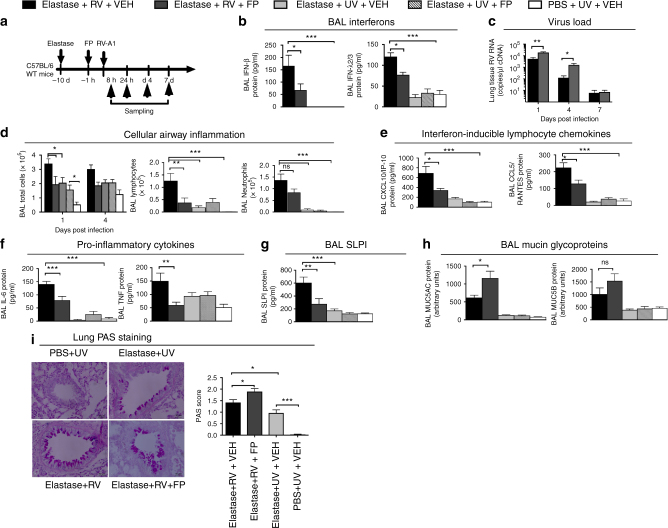
Fluticasone propionate impairs antiviral immunity and enhances mucus secretion in a mouse model of COPD exacerbation. **a** C57BL/6 mice were treated intranasally with a single dose of elastase or PBS as control. Ten days later, mice were treated intranasally with fluticasone propionate (20 μg) and challenged with rhinovirus (RV)-A1 or UV-inactivated RV-A1 (UV). **b** IFN-β and IFN-λ2/3 proteins were measured in bronchoalveolar lavage (BAL) by ELISA at 24 h post infection. **c** Rhinovirus RNA copies in lung tissue were measured by quantitative PCR. **d** Total cell numbers at days 1 and 4, lymphocyte numbers at day 4 and neutrophil numbers at day 1 post infection in BAL were enumerated by cytospin assay. **e** CXCL10/IP-10 and CCL5/RANTES proteins at 24 h and **f** IL-6 and TNF proteins at 8 h post infection were measured in BAL by ELISA. **g** Secretory leucocyte protease inhibitor (SLPI) protein at 24 h and **h** MUC5AC and MUC5B proteins at day 7 post infection were measured in BAL by ELISA. **i** At day 4 after RV challenge, lungs were formalin-fixed, paraffin-embedded and stained with periodic acid-Schiff (PAS). Left: Representative images of mice treated with PBS + UV, elastase + UV, elastase + RV and elastase + RV + FP are shown. Scale bars: 50 μm, Magnification, ×400. Right: Scoring for PAS-positive mucus-producing cells. Data represent mean (±SEM) of five–eight mice per treatment group, representative of at least two independent experiments. Data were analysed by one- or two-way ANOVA with Bonferroni post test. ns non-significant. *p < 0.05, **p < 0.01, ***p < 0.001

We investigated the effects of FP on RV-induced exacerbation of COPD-like disease in vivo in a mouse model of RV-exacerbated elastase-induced emphysema (Fig. 6a) in which many of the features of human virus-induced COPD exacerbations are recapitulated^25^. FP suppressed RV induction of IFN-β and IFN-λ2/3 proteins in BAL of elastase-treated mice (Fig. 6b) and increased virus load at days 1 and 4 post infection (Fig. 6c).

FP suppressed RV induction of BAL total cells and lymphocytes but had no significant effect on neutrophils (Fig. 6d). FP also suppressed RV induction of the lymphocyte chemokines CCL5/RANTES and CXCL10/IP-10 in BAL (Fig. 6e) and additionally suppressed RV induction of IL-6, TNF and SLPI (Fig. 6f, g). FP also enhanced RV induction of BAL MUC5AC protein (Fig. 6h) and mucus staining in lung sections (Fig. 6i), but there was no significant effect on BAL MUC5B protein levels (Fig. 6h).

### Adverse effects of ICS use during human COPD exacerbation

**Fig. 7:**
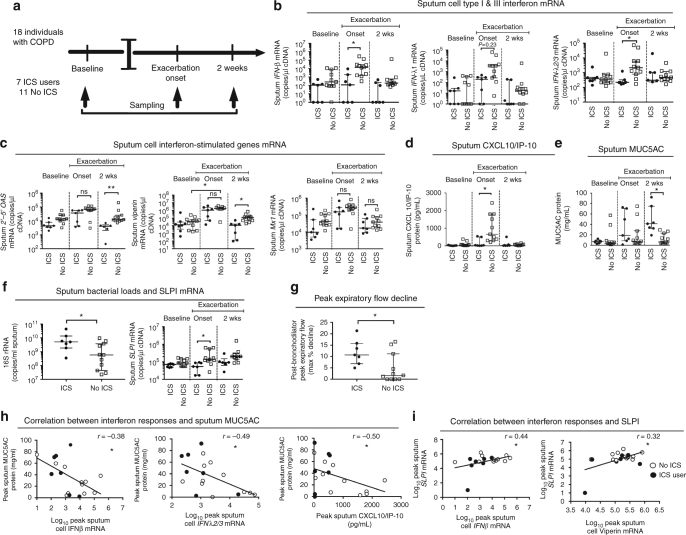
Inhaled corticosteroid use is associated with adverse effects during virus-induced COPD exacerbations. **a** Patients with COPD were monitored prospectively. Sputum samples were taken during stable state (baseline), at presentation with exacerbation associated with positive virus detection and 2 weeks during exacerbation. **b** *IFNβ*, *IFNλ1* and *IFNλ2/3* and **c** *2*′*–5*′*OAS, viperin* and *Mx1* mRNA expression in sputum cells was measured by quantitative PCR. **d** CXCL10/IP-10 protein, **e** MUC5AC protein was measured in sputum supernatants by ELISA. **f** Bacterial copy number of 16S rRNA in sputum at 2 weeks was measured by quantitative PCR. *SLPI* mRNA expression in sputum cells was measured by quantitative PCR. **g** Maximum post-bronchodilator peak expiratory flow rate % decline from baseline during exacerbation. **h** Correlation between peak sputum cell *IFNβ IFNλ2/3* mRNA expression and sputum CXCL10/IP-10 protein concentrations with peak sputum MUC5AC protein. **i** Correlation between peak sputum cell *IFNβ* and *Viperin* mRNAs expression with sputum cell *SLPI* mRNA expression. In **b**–**g**, data represent median (±IQR) per group. In **b**–**e** and **f** (right panel), data were analysed by Kruskal–Wallis test with Dunn’s post test. In **f** (left panel) and **g**, data were analysed by Mann–Whitney *U-*test. In **h**, **i**, correlation analysis used was non-parametric (Spearman’s correlation) performed on ICS users and ICS non-users pooled into a single group. ns non-significant; *p < 0.05; **p < 0.01, ***p < 0.001

To confirm the clinical relevance of our findings, we measured antiviral responses in sputum samples from a cohort of 40 patients with COPD, in which 18 episodes of virus-induced COPD exacerbations occurred (rhinovirus *n* = 11, coronavirus *n* = 4, RSV *n* = 2, influenza *n* = 1). Samples were assessed in 36 patients who had sufficient sample for evaluation at clinical stability (baseline) and in exacerbating patients at exacerbation onset and 2 weeks during exacerbation (Fig. 7a). Patients were stratified according to current use (*n* = 7) or non-use (*n* = 11) of ICS, baseline characteristics of these subgroups are in Table 2. ICS users had suppressed baseline sputum cell *IFNβ* expression versus ICS non-users with no significant differences observed for *IFNλ1* and *IFNλ2/3* (Supplementary Fig. 7a).

**Table 2:** Characteristics of patients with COPD presenting with virus-positive exacerbations, stratified according to current ICS use

	ICS users (*n* = 7)	ICS non-users (*n* = 11)	*p* Value
Age (median (IQR))	68 (63–74)	65 (45–71)	0.71
Male	4 (57.1%)	7 (63.6%)	1.0
GOLD stage (median (IQR))	2 (1.5–2)	2 (2–2.5)	0.46
Current smoker	3 (42.8%)	2 (18.2%)	0.33
Pack-year history (median (IQR))	40 (35–47.5)	50 (47.5–53)	0.12
Body mass index (median (IQR))	27.4 (26.1–28.4)	28.0 (23.1–32.9)	1.0
Ischaemic heart disease	0 (0%)	1 (9.1%)	1.0
Diabetes mellitus	1 (14.3%)	1 (9.1%)	1.0
Cerebrovascular disease	1 (14.3%)	0 (0%)	0.39
Prior annual exacerbation frequency (median (IQR))	3 (0.5–3.5)	2 (1–2)	0.67
Prior influenza vaccination	6 (85.7%)	8 (72.7%)	1.0
Beta agonist inhaler use	5 (71.4%)	8 (72.7%)	1.0
Anti-muscarinic inhaler use	4 (57.1%)	7 (63.6%)	1.0
Prednisolone initiated at exacerbation	2 (28.6%)	1 (9.1%)	0.53
Antibiotics initiated at exacerbation	4 (57.1%)	4 (36.4%)	0.63

Categorical variables were compared using the *χ*^2^ test. Continuous variables were compared using the Mann–Whitney *U*-test

IQR, interquartile range

In keeping with prior findings in vitro and in animal models, ICS users had suppressed sputum cell *IFNβ* and *IFNλ2/3* expression at exacerbation onset (Fig. 7b). Evaluation of mRNA expression as fold-change from baseline also demonstrated significant suppression in ICS users for *IFNλ2/3* (Supplementary Fig. 7b). As current smoking can affect antiviral responses^26^, we also carried out a sub-analysis excluding current smokers. In this subgroup, ICS users had significantly suppressed sputum cell *IFNλ2/3* (Supplementary Fig. 7c).

Sputum cell expression of *2*′*–5*′*-OAS* and *viperin* at 2 weeks was also significantly suppressed in ICS users versus non-users with no difference observed for *Mx1* (Fig. 7c). ICS users also had suppressed sputum supernatant CXCL10/IP-10 protein concentrations at exacerbation onset (Fig. 7d) and enhanced sputum MUC5AC glycoprotein concentrations at 2 weeks (Fig. 7e).

Secondary bacterial infection occurs following experimental RV infection in COPD subjects who were not taking ICS, with peak bacterial load occurring at day 15 post RV infection^14^. To determine if ICS use was associated with increased secondary bacterial infection in COPD, we evaluated bacterial load by 16S qPCR at the similar time point of 2 weeks post-exacerbation onset and found a significant increase in ICS users versus non-users (Fig. 7f). In keeping with effects of FP in mice, ICS users also had suppressed sputum cell *SLPI* expression at exacerbation (Fig. 7f).

ICS use during virus-induced COPD exacerbation was also associated with a greater maximum percentage decline from baseline in post-bronchodilator peak expiratory flow in ICS users versus ICS non-users (Fig. 7g).

### Correlation of IFNs with MUC5AC or SLPI during exacerbation

Consistent with our findings that type I IFN negatively regulates mucin production during RV infection in mice, we observed that peak sputum cell expression of *IFNβ*, *IFNλ2/3* and peak sputum concentrations of CXCL10/IP-10 protein negatively correlated with sputum MUC5AC during virus-induced COPD exacerbation (Fig. 7h). Similarly, in keeping with our findings in mice that interferon responses are also involved in SLPI production during virus infection, we found that peak sputum cell expression of *IFNβ* and *viperin* positively correlate with *SLPI* expression (Fig. 7i).

## Discussion

Using models of RV infection we have shown that FP, a commonly used ICS in man, suppresses critical antiviral immune responses, leading to impaired lung virus control and previously unrecognised adverse effects of mucin enhancement and impairment of antibacterial immunity, effects that were ameliorated by recombinant IFN-β administration and thus related to IFN suppression associated with FP administration. We importantly show that these adverse effects of FP also occur during COPD exacerbations, thus confirming relevance and importance in the context of human disease.

In primary RV infection models, FP suppressed induction of type I and type III IFNs by RV and delayed virus clearance in vivo. These data extend observations from previous studies which reported suppressed IFN responses to virus infection associated with corticosteroid administration in peripheral blood mononuclear cells^15^, fibroblasts^16^ and airway epithelial cells^16,27^. An intact type I IFN response is important for effective lung virus control and we have previously reported that *IFNAR1*^−/−^ mice exhibit increased virus loads when infected with RV^20^. In addition to showing impaired ex vivo IFN responses^1,8,28^, patients with COPD experimentally infected with RV have increased airway virus loads compared to controls without airway obstruction^1,29^, thereby indicating that IFN responses are also an important determinant of virus control in man. Our finding that FP administration in the mouse model delayed virus clearance in vivo is supported by human studies that have reported increased viral titres associated with systemic corticosteroid administration during RV challenged healthy subjects^30^ and increased RV shedding with intranasal FP administration during naturally occurring colds^31^, although conflicting data exists with another study showing only a trend towards increased virus shedding following combined treatment with oral and intranasal corticosteroid during experimental RV challenge^32^. Although the effect sizes observed in our mouse model were relatively small, we observe delayed virus clearance in an animal model where limited replication occurs^33^. In human RV infection, where replication is more robust and of longer duration^1^, ICS may be expected to have even more pronounced effects. However, we acknowledge that the mouse model of lung RV infection does not accurately model the sustained nasal viral replication seen in man. This restriction in viral replication means that our in vivo data showing ICS-mediated increase in virus loads should be interpreted with caution and further conclusive evidence on the effects of ICS on virus replication is required using ICS administration during experimental virus challenge in COPD patients.

We additionally identified which components of viral RNA recognition and/or IFNAR signalling pathways were disrupted by ICS using in vitro methods with specific PRR-activating viral RNA surrogates. In the experimental approach used, FP administration had no effect on ISG expression nor STAT1 and STAT2 phosphorylation, following stimulation with recombinant IFN-β, thereby suggesting that inhibitory effects of FP may be confined to initial virus-sensing pathways and not through effects on autocrine amplification via IFNAR. We have previously shown that induction of IFN by RV infection occurs initially via the endosomal PRR TLR3 and later via the inducible cytoplasmic helicases RIG-I and MDA-5^23^. In the current study, FP suppressed TLR3-mediated IFN expression, suggesting that corticosteroid inhibition of IFN production occurs through an early effect on the initial endosomal sensing of RVs. In keeping with this finding, we observed that FP suppressed *IFNβ* promoter activity induced by TRIF, the adaptor molecule utilised by TLR3 to stimulate type I IFN expression. We additionally evaluated the effect of FP administration on stimulation of airway epithelial cells by agonists for the cytosolic PRRs downstream of TLR3 and surprisingly found a differential effect with suppression of RIG-I-induced, but no effect on MDA-5-induced, IFN expression. Additionally, FP suppressed *IFNβ* promoter activity induced by MAVS, the adaptor molecule that is utilised by both MDA-5 and RIG-I to stimulate IFN expression. The reason for the discrepancy between the effect of FP on pathways mediated by the cytoplasmic receptors RIG-I and MDA-5 (which share many similarities) is unclear. It is possible that differences exist between how the adaptor molecule MAVS is utilised by MDA-5 and RIG-I to stimulate IFN production. Nonetheless, our data suggest that FP may exert its inhibitory effects on IFN induction through inhibition of the TLR3 virus-sensing pathway. This is consistent with a study which reported that FP suppressed poly(I:C) induction of CXCL10/IP-10 in BEAS-2B cells^34^. However, it should be noted that, although the selectivity of agonists used is supported by previous studies^35,36^, we cannot exclude that ligands employed in our studies do not trigger additional PRRs to those stated. Further evidence that ICS affect IFN signalling was shown in our mouse model, where FP administration suppressed activation of lung IRF-3, a signalling intermediate of the innate response. However, it should be noted that any nuclei obtained using our methods will be from multiple cell types, so it is possible that these data may be explained by effects of ICS on cellular composition.

Adaptive immune responses are important for both virus clearance and prevention of reinfection^37–40^ and we observed that FP administration prior to RV infection in mice led to suppressed cellular adaptive responses and impaired production of RV-specific IgG and neutralising antibodies. Our finding that FP impairs production of antibody responses to RV infection in the mouse model suggests that use of these commonly prescribed inhalers during COPD exacerbations could impair natural protective responses against future RV infections and thus limit long-term efficacy of ICS in reduction of exacerbations. This is supported by a previous study which reported that patients with exacerbation-prone COPD have lower RV-specific IgG1 antibody levels during stable state than non-exacerbating patients^41^, suggesting that deficient humoral immune responses to RV might be an important determinant of exacerbation risk.

RV induction of the major airway mucins MUC5AC and MUC5B was also enhanced by FP administration in the mouse model. Impaired mucociliary clearance with mucus hypersecretion is a feature of stable disease in COPD and likely contributes to airflow limitation, accelerated lung function decline and increased morbidity^42,43^. Additionally, lung MUC5AC overexpression has been reported in cases of fatal asthma and is thus associated with adverse outcomes^44^. RV infections can stimulate mucus production from airway epithelium and thereby potentiate sputum production during acute exacerbations^45,46^ and our finding that FP administration diminishes control of RV infection leading to increased mucin expression further suggests that use of these therapies may worsen mucus hypersecretion, leading to enhanced and/or prolonged symptoms and delayed recovery from exacerbation.

Administration of recombinant IFN-β in combination with FP and RV infection in mice reconstituted suppressed innate responses and improved virus clearance, suggesting that FP suppression of IFN responses plays a major mechanistic role in the adverse effects on virus control. RV-infected mice given steroid and then treated with IFN-β also had reduced MUC5AC expression compared with FP-treated RV-infected mice, which further supports the hypothesis that suppression of IFN underlies the enhanced MUC5AC production associated with FP administration. We additionally observed that *IFNAR1*^−/−^ mice which have diminished production of type I IFN in response to RV infection^20^ also have increased BAL MUC5AC, providing further evidence that type I IFN negatively regulates mucin production. Similar observations have been previously reported in *IFNAR1*^−/−^ mice infected with *Cryptococcus neoformans*^47^.

The association between ICS and pneumonia risk in COPD was first reported in 2007^9^ and has now been confirmed in numerous studies. Based on this perceived risk, some clinicians are now questioning the uncritical use of these widely prescribed therapies in COPD with ongoing evidence of practice change^48^. Remarkably, an underlying mechanism for this effect has never been identified. We have previously reported that experimental RV challenge induces secondary bacterial infection in COPD, through virus-induced suppression of the AMPs SLPI and elafin^14^. In the current studies, in addition to suppression of IFN, FP also suppressed RV induction of a number of airway antibacterial responses, including SLPI, accompanied by increases in bacterial load. Recombinant IFN-ß administration reversed suppression of SLPI and reduced increased bacterial loads associated with FP administration without affecting suppression of other antibacterial responses. Combined, these data suggest that FP may potentially increase risk of virus-induced secondary bacterial pneumonia through suppression of SLPI and that recombinant IFN therapy may prevent increased bacterial loads following RV infection. We speculate that this could be one mechanism explaining the increased pneumonia risk associated with ICS use in COPD^9,13^ and is consistent with previous data showing that higher RV loads are associated with increased risk of secondary bacterial infection in COPD^14^ and that ICS-related pneumonia episodes typically follow protracted symptomatic exacerbations^12^.

Some studies have previously suggested that corticosteroids may enhance SLPI production by bronchial epithelium in vitro^49,50^, but these studies were carried out in models of stable rather than exacerbated disease and conflicting data exists on the effects of corticosteroids in other cell types, such as THP-1 monocytes, where suppression rather than enhancement has been observed^51^. During virus infection, we clearly demonstrate in a range of models that FP has a suppressive effect on airway SLPI production. Since STAT1 has previously been shown to regulate SLPI expression^52^, FP likely impairs SLPI production during RV infection by suppressing PRR-induced IFN production thereby reducing subsequent IFNAR-mediated STAT1 activation. This mechanism is supported by our finding that *IFNAR1*^*−/−*^ mice also had reduced RV induction of SLPI. Additional evidence for the importance of deficient SLPI being an important mechanism for bacterial infection in COPD is provided by studies that have shown reduced airway SLPI in frequent (notably, of whom a greater proportion was taking ICS) versus infrequent COPD exacerbators^53^ and in patients with positive bacterial culture at exacerbation^54,55^.

Collectively, our studies using recombinant IFN administration in mice and RV infection in the *IFNAR1*^−/−^ mouse strain identify IFN as a central regulator of antibacterial responses and mucus production during RV infection. In a clinical trial in asthma patients taking ICS who developed a cold^56^, IFN-β therapy resulted in a trend towards reduced virus loads, with clinical benefit in patients with moderate/severe disease^56^. Our finding that IFN-β administration in the mouse model improved virus control, augmented antibacterial immunity and reversed the enhanced MUC5AC expression associated with FP treatment, adds further weight to the potential benefit of inhaled interferon as a treatment for virus-induced exacerbations of airway disease. Thomas et al. reported that administration of IFN-α and IFN-λ may similarly ameliorate ICS impairment of lung influenza virus control in mice^16^, although inhaled IFN-β is the therapy that is in clinical development and therefore of greatest current interest. Inhaled IFN-β has previously been shown to be safe in patients with asthma, and dedicated studies are required to investigate its utility in COPD.

It should be noted that the overall exacerbation reduction associated with ICS use in COPD outweighs the potential risk of pneumonia^57^. Our data showing that FP suppresses virus-induced airway inflammation supports the beneficial effects associated with use of these therapies. It is plausible that the adverse effects of ICS on host-defence reported in the current study might occur more frequently in the context of more severe infection, leading to unfavourable skewing of the risk/benefit ratio. Future development of selective steroids that retain potentially beneficial anti-inflammatory effects without having adverse effects on antiviral immunity could provide more effective approaches.

We additionally confirmed that the adverse effects of FP identified in our primary infection models also occur in models of COPD exacerbation. Evaluation of whether these detrimental effects occur in COPD is important because COPD is reported to be a relatively steroid-resistant disease^58^ and therefore it is feasible that steroid-induced adverse effects may not be observed in this context. In primary COPD AECs, FP suppressed RV induction of IFN responses and also enhanced mucus production. We confirmed these findings in vivo using a mouse model of COPD exacerbation^25^, to similarly demonstrate that FP suppresses RV-induced IFN responses leading to delayed virus clearance and mucus hypersecretion in vivo. Thus, FP administration in both in vitro and in vivo COPD models was associated with suppressed IFN responses, confirming that these adverse effects still occur in steroid-resistant disease models, and the increased virus load and mucus production observed in vivo could impact adversely on exacerbation severity. The elastase mouse model of COPD reproduces many of the immunopathological features of human COPD exacerbation^25^, but it is well recognised that existing models cannot completely recapitulate the complexities of human COPD, which occurs in genetically susceptible individuals following prolonged cigarette smoke exposure. Elastase administration represents a synthetic model of COPD and further animal studies using alternative modelling approaches, such as smoke exposure or genetic manipulation, are required to confirm our findings.

We confirmed the direct relevance of our findings to use of ICS in a clinical setting through analysis of sputum samples from patients with COPD presenting with virus-associated exacerbations. ICS use was associated with suppressed IFN responses at exacerbation with a concomitant increase in MUC5AC levels. In keeping with our findings in the mouse RV infection model and in support of our hypothesis that ICS-mediated suppression of SLPI promotes increased pneumonia risk in COPD, we also observed suppressed sputum *SLPI* mRNA and increased bacterial loads in ICS users. These data suggest that our prior in vitro and in vivo mouse model findings could be applicable in the context of human COPD exacerbations and provide preliminary clinical evidence that use of ICS may have adverse effects when used in exacerbating patients. Further studies using larger numbers of COPD subjects will be needed to confirm these data. A negative correlation between interferon responses and MUC5AC concentrations and positive correlation between interferon responses and SLPI expression was also observed, further supporting our conclusion that type I IFN plays a central role in regulation of mucus secretion and antimicrobial immunity during exacerbations.

**Fig. 8:**
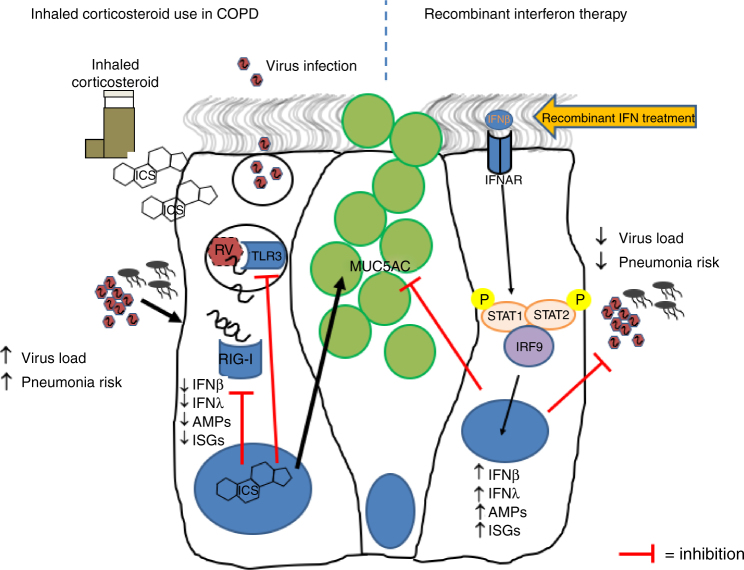
Schematic representation showing proposed adverse consequences of inhaled corticosteroid use during exacerbations. ICS use impairs type I interferon (IFN) production through effects on TLR3 and RIG-I virus-sensing pathways. Suppressed IFN leads to delayed virus clearance, mucus hypersecretion, deficient antimicrobial peptide responses and increased bacterial loads thereby increasing risk of secondary bacterial pneumonia. Use of recombinant IFN-β therapy bypasses this blockade and restores interferon responses, antimicrobial immunity and reduces mucus secretion, potentially leading to reduced exacerbation severity and lessened risk of pneumonia in COPD

In summary, these studies demonstrate that inhaled corticosteroid use suppresses antiviral immune responses, accompanied by adverse effects on mucin production and antibacterial immunity during virus-induced COPD exacerbations. The role of recombinant interferon as an adjunctive therapy to negate the adverse effects of ICS in COPD warrants further exploration. A schematic representation showing the proposed adverse consequences of inhaled corticosteroid use during exacerbations and potential beneficial effects of interferon therapy are shown in Fig. 8. Future development of novel selective steroids that retain beneficial anti-inflammatory effects but lack adverse effects on antimicrobial immunity and mucin production could be more effective for treatment/prevention of COPD exacerbations and thus be of greater benefit in clinical practice.

## Methods

### Cells and viruses

BEAS-2B cells (European Collection of cell cultures) were cultured in RPMI 1640 medium with 10% foetal calf serum (FCS). Rhinovirus serotype A1 was propagated in Ohio HeLa cells (European Collection of cell cultures) by standard methods and inactivated by exposure to UV light at 1200 mJ/cm^2^ for 30 min. For in vivo use in mouse models, virus was purified using precipitation in 7% polyethylene glycol 6000 (Sigma-Aldrich), 0.5 M NaCl, followed by filtration using a 0.2 μm syringe filter and buffer exchange and concentration in an Amicon Ultra-15 centrifugal filter unit (100 KDa nominal molecular weight limit) (Millipore, USA)^33,59^.

### Virus infection and treatment with FP and receptor agonists

BEAS-2B cells were cultured to 80% confluence in 12-well plates. Cells were treated with 1 or 10 nM fluticasone propionate (Sigma-Aldrich) or diluent alone (mock) for 1 h. Cultures were subsequently stimulated with 0.2 ml of RV-A1 (MOI 2) or 5 μg/ml TLR3 agonist (poly(I:C); Invitrogen), 250 ng/ml MDA-5 agonist (transfected poly(I:C); high MW form which is specific for MDA-5, as previously described^35^, Invivogen), 0.5 µg/ml RIG-I agonist (5′ triphosphate double-stranded RNA (5′ ppp-dsRNA), previously shown to be a specific ligand for RIG-I^36^; Invivogen) or 2.5 ng/ml recombinant IFN-β (R&D Systems) as appropriate for 1 h with shaking at room temperature. Viruses or agonists were then removed and replaced with 1 ml media and incubated for 24 h at 37 °C.

### Transient transfections of BEAS-2B cells with plasmid DNA

BEAS-2B cells were cultured to 80% confluence in 12-well plates. A reaction mix of 0.65 μg/well constitutionally active TRIF or MAVS (ΔTRIF or ΔMAVS, Invivogen) or pUNO1 control vector (Invivogen), 0.25 μg promoter–reporter plasmid (*IFNβ*-luciferase, a gift from T. Fujita, University of Osaka, Japan), 0.1 μg Renilla plasmid (Promega) and 3 µl Superfect (Qiagen, Crawley, UK) was diluted in serum-free RPMI and incubated for 15 min at room temperature. The mix was then diluted 1:5 with serum-free RPMI. Cells were washed twice with PBS followed by addition of 475 ml of the mix per well. Cells were then incubated at 37 °C for 3 h with 5% CO_2_. Three hours after transfections, cells were treated with FP at 1 and 10 nM concentrations, as described above and lysates were collected 24 h post transfection. Relative light units were assessed using a Dual Luciferase kit (Promega).

### Treatment and infection of primary bronchial epithelial cells

Primary bronchial epithelial cells were obtained from patients with GOLD stage 3 COPD bronchoscopically. All subjects gave informed written consent and the study protocol was approved by the Hunter New England Human Research Ethics Committee (05/08/10/3.09). Primary cells were grown in complete BEGM (Lonza) with growth factor supplements in submerged monolayer culture and then seeded at 2 × 10^5^ cells in transwells in a 12-well plate (Corning) with ALI-initial media comprised of 50%BEBM/50%DMEM containing 0.1% hydrocortisone, 0.1% bovine insulin, 0.1% epinephrine, 0.1% transferrin, 0.4% bovine pituitary extract (all from Lonza) and ethanolamine (final concentration 80 μM), MgCl_2_ (final concentration 0.3 mM), MgSO_4_ (final concentration 0.4 mM), bovine serum albumin (final concentration 0.5 mg/ml), amphotericin B (final concentration 250 μg/ml), all-trans retinoic acid (30 ng/ml) and 2% penicillin streptomycin with 10 ng/ml recombinant human epithelial growth factor (rhEGF) until confluent (at least 3 days in both apical and basal compartments). Once confluent, rhEGF concentration was changed to 0.5 ng/ml during the ALI phase for differentiation in the basal compartment (beneath the transwell insert) without apical media until day 21 after initial seeding. At day 21 after initial seeding, basal media was replaced with media containing 1 or 10 nM fluticasone propionate, to appropriate wells. RV-A1 infection (MOI 0.1) was applied to the apical surface for 2 h in 250 μl BEBM minimal at 35 °C, after which, infection media was replaced with 500 μl fresh BEBM minimal for the remainder of the time course. Samples were collected at 24, 48, 72 and 96 h post infection. At each time point, apical media was removed from the cultures and stored for protein expression analyses and half of the transwell membrane was carefully cut from the inserts and collected into RLT buffer (Qiagen) containing 1% 2-mercaptoethanol for downstream molecular analyses by RT-qPCR while the remaining transwell membrane was fixed in 10% neutral-buffered formalin for 24 h for histological cross-sections to confirm differentiation and stained with periodic acid-Schiff (PAS) to assess mucus production.

### Rhinovirus infection and treatment of mice

In vivo protocols: Female mice (8–10 weeks of age) on a C57BL/6 background were used for all animal studies. Mice were purchased from Charles River Laboratories and housed in individually ventilated cages under specific pathogen-free conditions. All animal work was performed under the authority of the UK Home Office outlined in the Animals (Scientific Procedures) Act 1986 after ethical review by Imperial College London Animal Welfare and Ethical Review Body (project licence PPL 70/7234)

FP powder (Sigma-Aldrich) was resuspended at a concentration of 357 μg/ml in dimethyl sulfoxide (DMSO; Sigma-Aldrich) and then diluted 1:1000 in PBS. Mice were treated intranasally under light isoflurane anaesthesia with 50 μl of FP solution (equating to 20 μg FP dose) or vehicle DMSO diluted 1:1000 in PBS as control. One hour after FP or vehicle administration, mice were infected intranasally with 50 μl RV-A1 (5 × 10^6^ TCID_50_) or UV-inactivated RV-A1 control (as detailed above)^33^. In separate experiments, 1 h following RV-A1 or UV-RV-A1 challenge, mice were additionally treated intranasally with 50 μl of PBS containing 10^4^ units of recombinant IFN-β (R&D systems).

### Fluticasone administration in COPD exacerbation mouse model

Mice were treated intranasally under light isoflurane anaesthesia with 1.2 units of porcine pancreatic elastase (Merck) to induce emphysematous lung changes^25^, Ten days following elastase administration, mice were treated intranasally with 20 μg FP solution (prepared as detailed above), 1 h prior to intranasal infection with 50 μl RV-A1 (2.5 × 10^6^ TCID_50_, a 50% dose reduction from the primary RV infection model) or UV-inactivated RV-A1 control, as detailed above.

### Naturally occurring human COPD exacerbation cohort

A cohort of 40 COPD subjects was recruited to a study investigating naturally occurring exacerbations between June 2011 and December 2013. Subjects of all grades of COPD severity, confirmed by spirometry, were recruited and all treatments were permitted. All subjects gave informed written consent and the study protocol was approved by the East London Research Ethics Committee (Protocol number 11/LO/0229). All subjects had an initial baseline visit during clinical stability for medical assessment, peak expiratory flow rate measurement, spirometry (forced expiratory volume in 1 s (FEV1), forced vital capacity (FVC) and clinical sample collection.

Sputum induction and processing: Sputum was induced using pre-medication with 200 µg salbutamol via metered dose inhaler and large volume spacer. Four per cent saline was administered using a DeVilbiss UltraNeb99 ultrasonic nebuliser until an adequate sputum sample was obtained. Sputum was processed within 2 h of induction. Sputum plugs were selected from saliva by macroscopic inspection of the sample. An aliquot was selected and stored unprocessed at –80 °C. The remaining sample was weighed and 0.1% dithiothreitol (DTT) was added in the ratio 4 ml DTT to 1 g sputum. The mixture was then agitated and filtered. The same volume PBS was added, the filtrate centrifuged and the supernatant aliquotted and stored at −80 °C.

Virus detection: Viruses were detected in sputum using polymerase chain reaction. The following viruses were investigated: adenovirus, coronavirus, human bocavirus, human metapneumovirus, influenza (AH1, AH3, B), parainfluenza 1–3, picornavirus and respiratory syncytial virus. RNA was extracted using QIAamp viral RNA mini kit (Qiagen). For picornaviruses, 5 µl RNA was converted to cDNA by addition to a reaction mix containing 1.55 µl OL27 (Invitrogen), 1 µl 0.1 M DTT, 0.2 µl dNTPs (Invitrogen) and 0.25 µl (10,000U) reverse transcriptase (Invitrogen). A single round of PCR of 32 cycles was then used to detect picornaviruses in this cDNA. Differentiation of RVs from enteroviruses was achieved by restriction enzyme digestion of the PCR product.

For all other viruses, conversion to cDNA was achieved with 18 µl RNA added to a reaction mix containing 2.5 µl of random hexamer primers (0.5 µg/µl, Promega) and 6.5 µl of UHQ (nuclease-free water, Promega). This mixture was denatured at 70 °C for 10 min. After quenching on ice, 10 µl of 5× first-strand buffer, 6 µl of UHQ, 5 µl 0.1 M DTT, 1.25 µl dNTPs and 2 µl (200U) of reverse transcriptase (Qiagen) were added following by incubation at 37 °C for 1 h to yield cDNA. An aliquot of 4 µl of this cDNA was then used for each RT-PCR in the panel, performed using a 2720 Thermal Cycler (Applied Biosystems).

Follow-up: Subjects had repeat visits at three monthly intervals when clinically stable and were followed up for a minimum of 6 months. Subjects reported to the study team when they developed symptoms of an acute exacerbation defined using the East London cohort criteria^60^ and were seen within 48 h of symptom onset. Sputum samples were collected at the onset of exacerbation and at 2 weeks during exacerbation.

### Cell assays

In mouse models, BAL was performed by cannulation through the trachea and lavage with 1.5 ml sterile PBS. Cells were pelleted by centrifugation, resuspended in ACK buffer to lyse red cells, washed in PBS and resuspended in RPMI medium with 10% FBS. Cells were stained with Quik Diff (Reagena) for differential counting. For flow cytometry analysis, lung leukocytes were obtained from BAL and red cells were lysed with ACK buffer. BAL cells were stained with Live/Dead fixable dead cell stain (Life Technologies, Carlsbad, CA), incubated with anti-mouse CD16/CD32 (FC block; BD Biosciences) and subsequently with directly fluorochrome-conjugated monoclonal antibodies specific for CD3ε (clone 500 A; 2 1 µg/ml, BD Biosciences), CD69 (clone H1.2F3 1 µg/ml, BD Biosciences), CD4 (clone RM4-5 0.25 µg/ml; BD Biosciences), CD8a (clone 53–6.7; 0.5 µg/ml BD Biosciences) and NK1.1 (clone PK136;1 µg/ml, BD Biosciences). Data were acquired on an LSR II flow cytometer (BD Biosciences) and analysed using FlowJo software (version 10.0.6; Tree Star, Ashland, USA). Representative gating strategies used for analysis of cell surface staining are shown in Supplementary Fig. 8.

### Protein assays

Cytokine protein levels in mouse BAL, human sputum supernatants or cell supernatants from in vitro experiments were assayed using commercial 'duoset' enzyme-linked immunosorbent assay kits (R&D Systems, Abingdon, UK). MUC5AC and MUC5B proteins in BAL were measured after adhesion to a 96-well plate by allowing samples to evaporate at 37 °C overnight. Plates were washed three times between steps. Plates were blocked for 2 h with 2% bovine serum, albumin (BSA) in phosphate-buffered saline. For measurement of MUC5AC, the detection antibody used was biotinylated anti-MUC5AC (Thermo Scientific) at 400 ng/ml. For the MUC5B assay, detection antibody was mouse anti-MUC5B clone EH-MUC5Ba. Bound anti-MUC5B antibody was detected with peroxidase-conjugated goat anti-mouse IgG (Sigma-Aldrich). Standard curves for mucin ELISAs were generated by serial 1:2 dilutions of supernatants taken from ionomycin-stimulated H292 cells (MUC5AC) and BAL supernatants previously taken from ovalbumin-induced hyper-allergic mice (MUC5B).

Western blotting was used to evaluate proteins in BEAS-2B cell extracts. Cells were lysed in radioimmunoprecipitation (RIPA) buffer (Sigma-Aldrich) supplemented with protease (Roche) and phosphatase inhibitors (Sigma-Aldrich). Protein content was measured by the bicinchoninic acid assay (Thermo Scientific). Equal amounts of protein were loaded onto 4–12% Bis-Tris SDS-PAGE gels and transferred onto polyvinylidene difluoride (PVDF) membranes (both Life Technologies). Membranes were blocked with 5% BSA in Tris-buffered saline for 1 h at room temperature. The following primary antibodies were used: rabbit anti-pSTAT1 Y701 (1:1000 diluted; New England BioLabs), anti-pSTAT2 Y690 (1:1000, New England BioLabs), anti-STAT1 (1:1000 diluted; New England BioLabs) and anti-STAT2 (1:1000 diluted; New England BioLabs). Primary antibodies were incubated overnight at 4 °C with shaking and secondary antibody conjugated to horseradish peroxidase (1:5000 diluted; Jackson Immunoresearch) was incubated for 1 h at room temperature. Data were acquired using a Fusion FX7 image analyser (Vilber Lourmat). Uncropped western blot images are shown in Supplementary Fig 9.

For measurement of serum antibodies in the mouse model, peripheral blood was collected from the carotid arteries and serum RV-specific IgGs were measured by in-house enzyme-linked immunosorbent assay. Ninety-six-well plates were coated with purified RV1B, as used for in vivo infection, and incubated at 4 °C overnight, Plates were blocked with 5% milk in PBS-0.05% Tween 20 at room temperature. An aliquot of 50 µl per well of serum diluted 1:50 in 5% milk/PBS was then added with further 2 h incubation at room temperature. IgG detection antibodies were biotinylated rat anti-mouse IgG1 (0.5 µg/ml clone A85-1 BD Biosciences) and IgG2a (0.5 µg/ml clone R19-15. BD Biosciences).

To prepare lung nuclear extracts for measurement of transcription factor activation, the left lung was excised, immediately placed into a 1.7 ml tube and snap frozen in liquid nitrogen followed by storage at −80 °C. Lung tissue was manually homogenised while immersed in liquid nitrogen using a mortar and pestle and nuclear extracts prepared using a nuclear extract kit (Active Motif, La Hulpe, Belgium). Activation of transcription factors GR, NFκB p65 subunit and IRF-3 were assessed in mouse lung tissue using commercially available DNA binding assays (Active Motif) using 20 μg per well of nuclear protein.

### Neutralisation assays

Neutralisation of RV was measured in Ohio HeLa cells (UK Health Protection Agency General Cell Collection) grown until ~90% confluent on 96-well plates. Pooled sera for each given treatment group were incubated with purified RV-A1 or medium control at room temperature with shaking for 1 h and then added to HeLa cells with further incubation at 37 °C for 48–72 h. Protection from cytopathic effect was measured by crystal violet cell viability assay. Cells were stained with 0.1% (w/v) crystal violet (Sigma-Aldrich) at room temperature for 10 min. Cells were then washed with water, air-dried and crystal violet was solubilised with 1% sodium dodecyl sulphate (SDS) solution (w/v). Absorbance was measured by a Spectramax Plus plate reader at 560 nm.

### RNA extraction, cDNA synthesis and quantitative PCR

Total RNA was extracted from BEAS-2Bs, primary airway epithelial cells or sputum cells (RNeasy kit, Qiagen) and 2 µg was used for cDNA synthesis (Omniscript RT kit). For mouse models, total RNA was extracted from the right upper lobe of mouse lung and placed in RNA later, prior to RNA extraction and cDNA synthesis (as detailed above). Quantitative PCR was carried out using previously described specific primers and probes for each gene of interest^23,33^, and normalised to 18S rRNA^33^. Reactions were analysed using ABI 7500 Fast Real-time PCR system (Applied Biosystems, Carlsbad, CA).

### DNA extraction and bacterial 16S quantitative PCR

DNA extraction from human sputum and mouse lung tissue was performed using the FastDNA Spin Kit for Soil (MP Biomedicals, Santa Ana, USA), as per the manufacturer's instructions. Bead-beating was performed for two cycles of 30 s at 6800 rpm (Precellys, Bertin Technologies). Total 16s bacterial loads were measured using quantitative PCR, performed in triplicate using the Viia7 Real-Time PCR system (Life Technologies). In addition to template, each PCR reaction mix contained 0.2 μl of forward primer 520F (10 μm; 5′-AYTGGYDTAAAGNG-3′), 0.2 μl reverse primer 802R (10 μm; 5′-TACNVGGGTATCTAATCC-3′), 5 μl SYBR Fast Universal master mix (Kapa Biosystems) and 3.6 μl H_2_O. PCR cycling conditions were: 1 cycle of 90 °C for 3 min, followed by 40 cycles of 95 °C (20 s), 50 °C (30 s), 72 °C (30 s) and melt conditions of 1 cycle of 95 °C (15 s) and 1 cycle of 60 °C (1 min) followed by dissociation at 95 °C (15 s). For creation of the standard curve, a 1:10 dilution series (10^4^–10^9^ copies) of partial 16S rRNA gene of *Vibrio natriegens* DSMZ 759 (Deutsche Sammlung von Mikroorganismen, Braunschweig, Germany) from positions 27 to 1492 of the *Escherichia coli* reference cloned using TOPO TA cloning kit; (Invitrogen, ThermoFisher Scientific) was used.

### Statistical analyses

For mouse experiments, animals were studied in group sizes of five–eight mice per experimental condition and data are presented as mean ± SEM, representative of at least two combined independent experiments. Data were analysed using one- or two-way ANOVA with significant differences between groups assessed by Bonferroni’s multiple comparison test. In vitro BEAS-2B experiments were performed three–five times and data are expressed as mean ± SEM. Data were analysed by one- or two-way ANOVA test with differences between groups assessed Bonferroni’s multiple comparison test. In vitro primary airway epithelial cell experiments were performed with a group size of *n* = 9 patients and individual data points are shown. Data were analysed using one-way ANOVA with significant differences between groups assessed by Bonferroni’s multiple comparison test. Data from the human COPD cohort were analysed by using the Mann–Whitney *U*-test or Kruskal–Wallis test with Dunn’s post test to compare protein levels or mRNA expression between ICS users and non-users. Correlations between data sets were examined using Spearman’s rank correlation coefficient. All statistics were performed using GraphPad Prism 6 software. Differences were considered significant when *p* < 0.05.

### Data availability

The data supporting the findings of the study are available in this article and its Supplementary Information files, or from the corresponding author on request.

## Electronic supplementary material

Supplementary Information

## References

[1] Mallia P (2011). Experimental rhinovirus infection as a human model of chronic obstructive pulmonary disease exacerbation. Am. J. Respir. Crit. Care Med..

[2] Message SD (2008). Rhinovirus-induced lower respiratory illness is increased in asthma and related to virus load and Th1/2 cytokine and IL-10 production. Proc. Natl Acad. Sci. USA.

[3] Jackson DJ (2014). IL-33-dependent type 2 inflammation during rhinovirus-induced asthma exacerbations in vivo. Am. J. Respir. Crit. Care Med..

[4] Sykes A (2012). Rhinovirus 16-induced IFN-alpha and IFN-beta are deficient in bronchoalveolar lavage cells in asthmatic patients. J. Allergy Clin. Immunol..

[5] Wark PA (2005). Asthmatic bronchial epithelial cells have a deficient innate immune response to infection with rhinovirus. J. Exp. Med..

[6] Contoli M (2006). Role of deficient type III interferon-lambda production in asthma exacerbations. Nat. Med..

[7] Collison A (2013). The E3 ubiquitin ligase midline 1 promotes allergen and rhinovirus-induced asthma by inhibiting protein phosphatase 2A activity. Nat. Med..

[8] Hsu AC (2016). Impaired antiviral stress granule and IFN-beta enhanceosome formation enhances susceptibility to influenza infection in chronic obstructive pulmonary disease epithelium. Am. J. Respir. Cell Mol. Biol..

[9] Calverley PM (2007). Salmeterol and fluticasone propionate and survival in chronic obstructive pulmonary disease. N. Eng. J. Med..

[10] Wedzicha JA (2008). The prevention of chronic obstructive pulmonary disease exacerbations by salmeterol/fluticasone propionate or tiotropium bromide. Am. J. Respir. Crit. Care Med..

[11] Kardos P, Wencker M, Glaab T, Vogelmeier C (2007). Impact of salmeterol/fluticasone propionate versus salmeterol on exacerbations in severe chronic obstructive pulmonary disease. Am. J. Respir. Crit. Care Med..

[12] Calverley PM (2011). Reported pneumonia in patients with COPD: findings from the INSPIRE study. Chest.

[13] Drummond MB, Dasenbrook EC, Pitz MW, Murphy DJ, Fan E (2008). Inhaled corticosteroids in patients with stable chronic obstructive pulmonary disease: a systematic review and meta-analysis. JAMA.

[14] Mallia P (2012). Rhinovirus infection induces degradation of antimicrobial peptides and secondary bacterial infection in chronic obstructive pulmonary disease. Am. J. Respir. Crit. Care Med..

[15] Davies JM (2011). Budesonide and formoterol reduce early innate antiviral immune responses in vitro. PLoS ONE.

[16] Thomas BJ, Porritt RA, Hertzog PJ, Bardin PG, Tate MD (2014). Glucocorticosteroids enhance replication of respiratory viruses: effect of adjuvant interferon. Sci. Rep..

[17] Barnes PJ (2013). Corticosteroid resistance in patients with asthma and chronic obstructive pulmonary disease. J. Allergy Clin. Immunol..

[18] Mitani A, Ito K, Vuppusetty C, Barnes PJ, Mercado N (2016). Restoration of corticosteroid sensitivity in chronic obstructive pulmonary disease by inhibition of mammalian target of rapamycin. Am. J. Respir. Crit. Care Med..

[19] Singam R (2006). Combined fluticasone propionate and salmeterol reduces RSV infection more effectively than either of them alone in allergen-sensitized mice. Virol. J..

[20] Bartlett NW (2012). Defining critical roles for NF-kappaB p65 and type I interferon in innate immunity to rhinovirus. EMBO Mol. Med..

[21] Wang Q (2009). Role of double-stranded RNA pattern recognition receptors in rhinovirus-induced airway epithelial cell responses. J. Immunol..

[22] Voynow JA, Rubin BK (2009). Mucins, mucus, and sputum. Chest.

[23] Slater L (2010). Co-ordinated role of TLR3, RIG-I and MDA5 in the innate response to rhinovirus in bronchial epithelium. PLoS Pathog..

[24] Seth RB, Sun L, Ea CK, Chen ZJ (2005). Identification and characterization of MAVS, a mitochondrial antiviral signaling protein that activates NF-kappaB and IRF 3. Cell.

[25] Singanayagam A (2015). A short-term mouse model that reproduces the immunopathological features of rhinovirus-induced exacerbation of COPD. Clin. Sci..

[26] Wu W (2016). Human primary airway epithelial cells isolated from active smokers have epigenetically impaired antiviral responses. Respir. Res..

[27] Skevaki CL (2009). Budesonide and formoterol inhibit inflammatory mediator production by bronchial epithelial cells infected with rhinovirus. Clin. Exp. Allergy.

[28] Hsu AC (2017). MicroRNA-125a and -b inhibit A20 and MAVS to promote inflammation and impair antiviral response in COPD. JCI Insight.

[29] Footitt J (2016). Oxidative and nitrosative stress and histone deacetylase-2 activity in exacerbations of COPD. Chest.

[30] Gustafson LM, Proud D, Hendley JO, Hayden FG, Gwaltney JM (1996). Oral prednisone therapy in experimental rhinovirus infections. J. Allergy Clin. Immunol..

[31] Puhakka T (1998). The common cold: effects of intranasal fluticasone propionate treatment. J. Allergy Clin. Immunol..

[32] Farr BM (1990). A randomized controlled trial of glucocorticoid prophylaxis against experimental rhinovirus infection. J. Infect. Dis..

[33] Bartlett NW (2008). Mouse models of rhinovirus-induced disease and exacerbation of allergic airway inflammation. Nat. Med..

[34] Matsukura S (2013). Basic research on virus-induced asthma exacerbation: inhibition of inflammatory chemokine expression by fluticasone propionate. Int. Arch. Allergy Immunol..

[35] Kato H (2008). Length-dependent recognition of double-stranded ribonucleic acids by retinoic acid-inducible gene-I and melanoma differentiation-associated gene 5. J. Exp. Med..

[36] Hornung V (2006). 5’-Triphosphate RNA is the ligand for RIG-I. Science.

[37] Barclay WS, al-Nakib W, Higgins PG, Tyrrell DA (1989). The time course of the humoral immune response to rhinovirus infection. Epidemiol. Infect..

[38] Parry DE (2000). Rhinovirus-induced PBMC responses and outcome of experimental infection in allergic subjects. J. Allergy Clin. Immunol..

[39] Alper CM (1998). Prechallenge antibodies moderate disease expression in adults experimentally exposed to rhinovirus strain hanks. Clin. Infect. Dis..

[40] McKinstry KK (2012). Memory CD4+ T cells protect against influenza through multiple synergizing mechanisms. J. Clin. Invest..

[41] Yerkovich ST (2012). Reduced rhinovirus-specific antibodies are associated with acute exacerbations of chronic obstructive pulmonary disease requiring hospitalisation. BMC Pulm. Med..

[42] Aikawa T (1989). Morphometric analysis of intraluminal mucus in airways in chronic obstructive pulmonary disease. Am. Rev. Respir. Dis..

[43] Vestbo J, Prescott E, Lange P (1996). Association of chronic mucus hypersecretion with FEV1 decline and chronic obstructive pulmonary disease morbidity. Copenhagen City Heart Study Group. Am. J. Respir. Crit. Care Med..

[44] Groneberg DA (2002). Expression of respiratory mucins in fatal status asthmaticus and mild asthma. Histopathology.

[45] Hewson CA (2010). Rhinovirus induces MUC5AC in a human infection model and in vitro via NF-kappaB and EGFR pathways. Eur. Respir. J..

[46] Zhu L (2009). Rhinovirus-induced major airway mucin production involves a novel TLR3-EGFR-dependent pathway. Am. J. Respir. Cell Mol. Biol..

[47] Sato K (2015). Cryptococcus neoformans infection in mice lacking type I interferon signaling leads to increased fungal clearance and IL-4-dependent mucin production in the lungs. PLoS ONE.

[48] Chalmers JD, Tebboth A, Gayle A, Ternouth A, Ramscar N (2017). Determinants of initial inhaled corticosteroid use in patients with GOLD A/B COPD: a retrospective study of UK general practice. NPJ Prim. Care Respir. Med..

[49] Abbinante-Nissen JM, Simpson LG, Leikauf GD (1995). Corticosteroids increase secretory leukocyte protease inhibitor transcript levels in airway epithelial cells. Am. J. Physiol..

[50] Usmani OS (2005). Glucocorticoid receptor nuclear translocation in airway cells after inhaled combination therapy. Am. J. Respir. Crit. Care Med..

[51] Kulkarni NN (2016). Glucocorticoid dexamethasone down-regulates basal and vitamin D3 induced cathelicidin expression in human monocytes and bronchial epithelial cell line. Immunobiology.

[52] Meyer M (2014). Regulation and activity of secretory leukoprotease inhibitor (SLPI) is altered in smokers. Am. J. Physiol. Lung Cell. Mol. Physiol..

[53] Gompertz S, Bayley DL, Hill SL, Stockley RA (2001). Relationship between airway inflammation and the frequency of exacerbations in patients with smoking related COPD. Thorax.

[54] Pant S (2009). Airway inflammation and anti-protease defences rapidly improve during treatment of an acute exacerbation of COPD. Respirology.

[55] Parameswaran GI, Sethi S, Murphy TF (2011). Effects of bacterial infection on airway antimicrobial peptides and proteins in COPD. Chest.

[56] Djukanovic R (2014). The effect of inhaled IFN-beta on worsening of asthma symptoms caused by viral infections. A randomized trial. Am. J. Respir. Crit. Care Med..

[57] Suissa S (2013). Number needed to treat in COPD: exacerbations versus pneumonias. Thorax.

[58] Ito K (2006). Histone deacetylase 2-mediated deacetylation of the glucocorticoid receptor enables NF-kappaB suppression. J. Exp. Med..

[59] Bartlett NW, Singanayagam A, Johnston SL (2015). Mouse models of rhinovirus infection and airways disease. Methods Mol. Biol..

[60] Donaldson GC, Seemungal TA, Bhowmik A, Wedzicha JA (2002). Relationship between exacerbation frequency and lung function decline in chronic obstructive pulmonary disease. Thorax.

