# Supplementary material for: Corticosteroid suppression of antiviral immunity increases bacterial loads and mucus production in COPD exacerbations
Source: Nat Commun. 2018 Jun 8;9:2229. doi: 10.1038/s41467-018-04574-1 (PMC5993715; doi:10.1038/s41467-018-04574-1)
Supplement: Supplementary file 1 — Supplementary Information [file 41467_2018_4574_MOESM1_ESM.pdf]

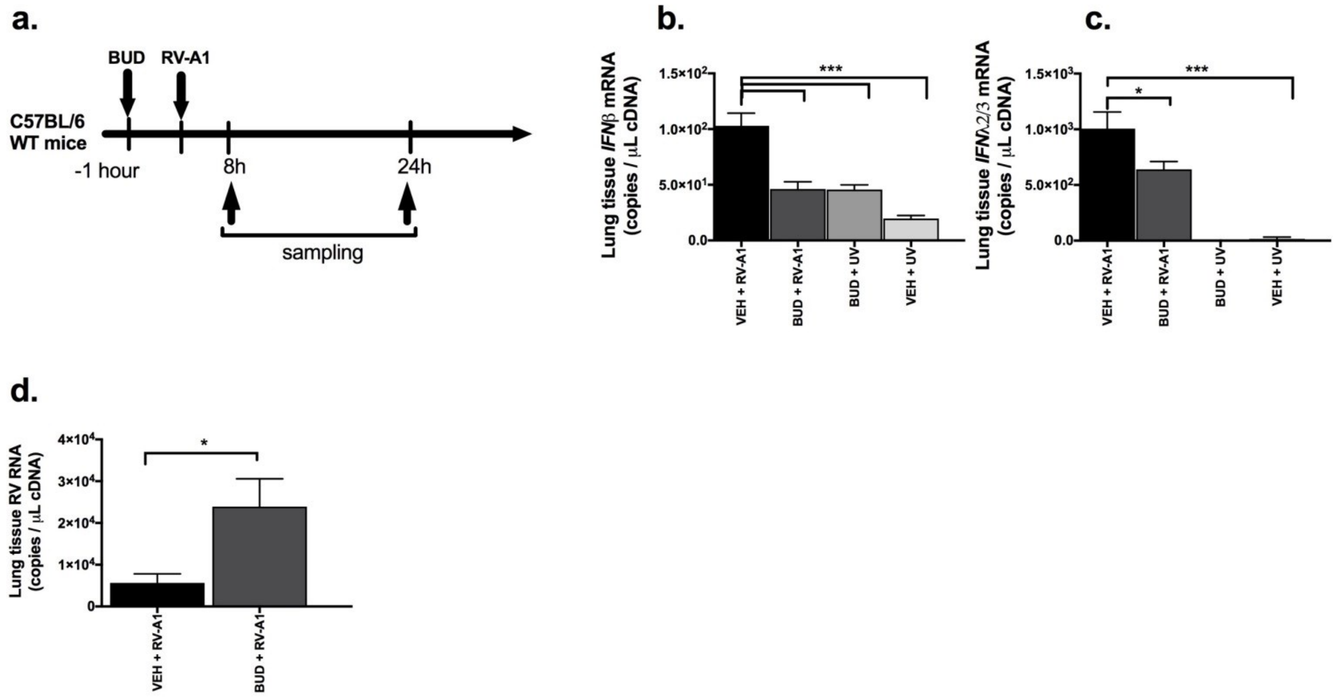

Supplementary fig 1: Budesonide suppresses innate anti-viral immune responses and increases rhinovirus loads in mice.
(a) C57BL/6 mice were treated intranasally with budesonide (20μg) or vehicle DMSO control and challenged intranasally with rhinovirus (RV)-A1 or UV-inactivated RV-A1 (UV) (b) *IFNβ* and (c) *IFNλ2/3* mRNAs in lung tissue at 8 hours post-infection were measured by quantitative PCR**.** (d) Rhinovirus RNA copies in lung tissue were measured by quantitative PCR**.** Data represents mean (+/-SEM) of 5 mice per treatment group, representative of at least two independent experiments. Data were analysed by one-way ANOVA with Bonferroni post-test. ns = non-significant. *p<0.05, ***p<0.001.

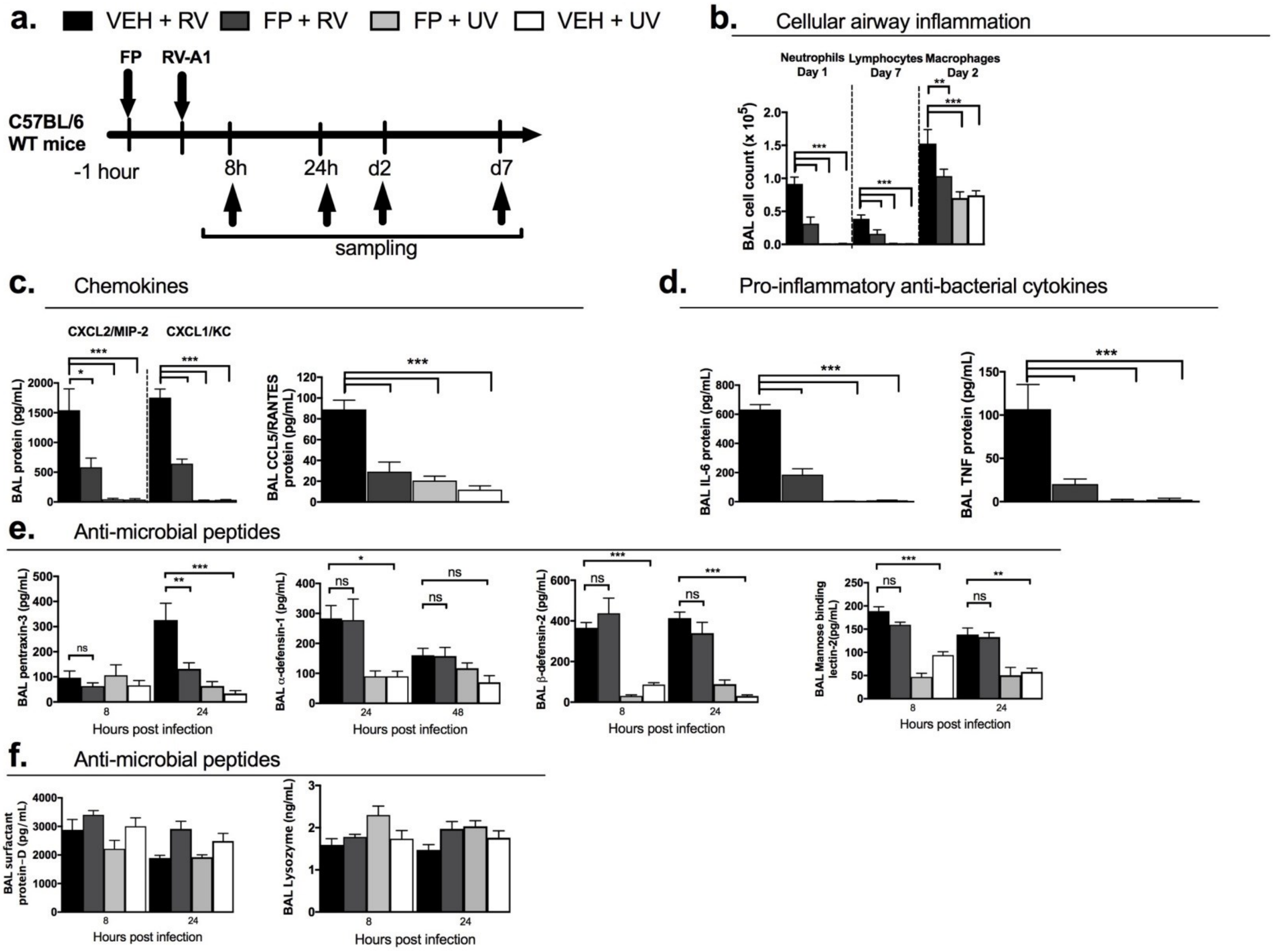

Supplementary Figure 2: Effect of Fluticasone propionate on rhinovirus-induced airway inflammation and anti-microbial peptides. (a) C57BL/6 mice were treated intranasally with fluticasone propionate (20μg) or vehicle DMSO control and challenged intranasally with rhinovirus (RV)-A1 or UV-inactivated RV-A1 (UV). (b) Neutrophils, lymphocytes and macrophages in BAL were enumerated by cytospin assay. (c) CXCL2/MIP-2, CXCL1/KC at 8 hours and CXCL10/IP-10 and CCL5/RANTES proteins in bronchoalveolar lavage (BAL) at 24 hours post-infection were measured by ELISA. (d) IL-6 and TNF proteins in BAL at 8 hours post-infection were measured by ELISA. (e) Anti-microbial peptides pentraxin-3, α-defensin 1, β-defensin 2, mannose binding lectin-2 and (f) surfactant protein-D and lysozyme in BAL were measured by ELISA.. Data represents mean (+/-SEM) of 5-8 mice per treatment group, representative of at least two independent experiments. Data were analysed by one-way ANOVA with Bonferroni post-test. ns = non-significant. *p<0.05, **p<0.01, ***p<0.001.

S

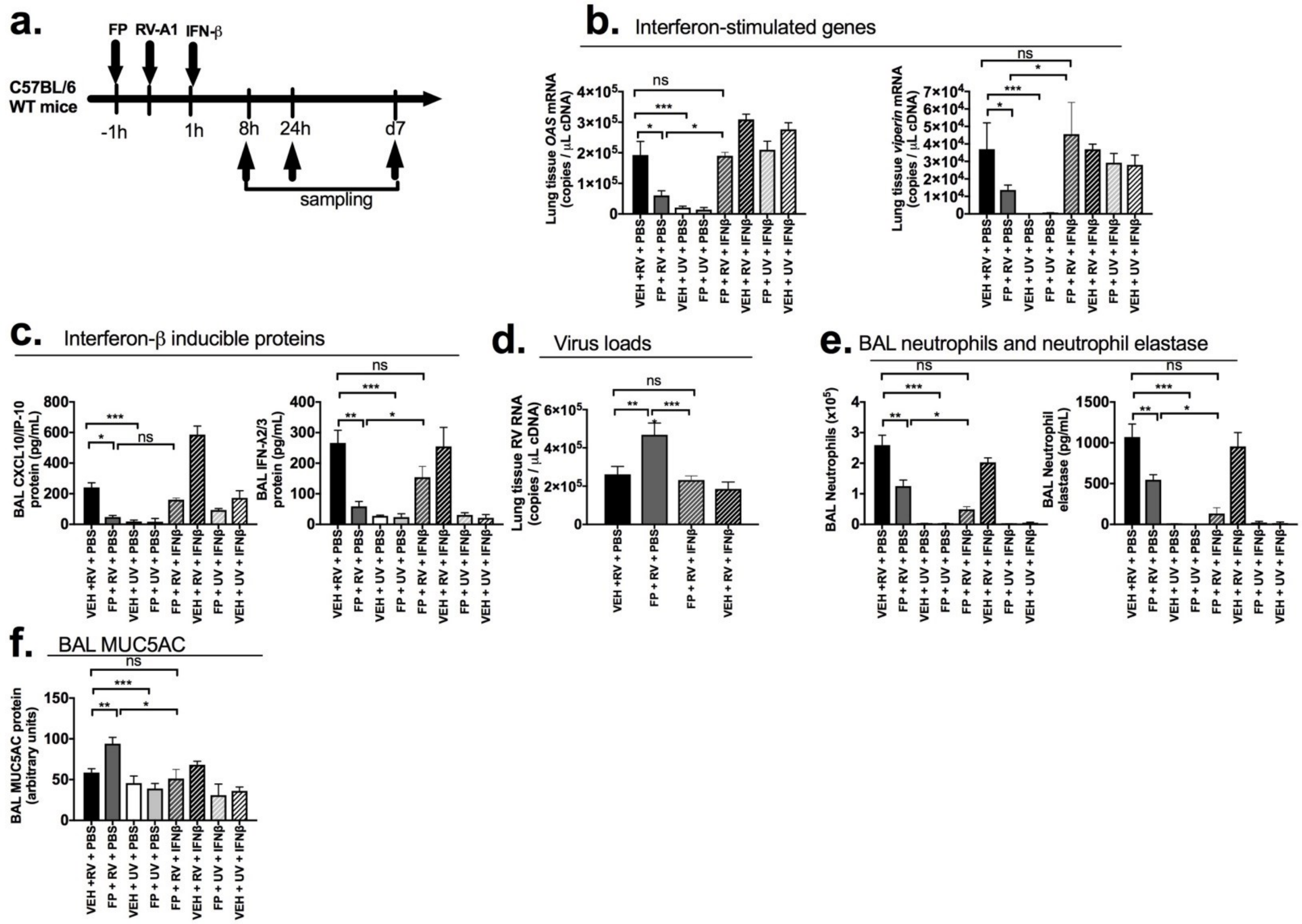

Supplementary Figure 3: Full dataset showing all control groups for recombinant interferon-β administration experiments in mice. (a) C57BL/6 mice were treated intranasally with fluticasone propionate (20µg) or vehicle DMSO control and challenged intranasally with rhinovirus (RV)-A1 or UV-inactivated RV-A1 (UV). One hour after RV challenge, mice were additionally treated intranasally with 10^4 units recombinant IFN-β. (b) 2'-5' *OAS* and *viperin* mRNAs in lung tissue were measured by quantitative PCR at 8 hours post-infection. (c) CXCL10/IP-10 and IFN-λ2/3 proteins were measured in bronchoalveolar lavage (BAL) by ELISA at 24 hours post-infection. (d) RV RNA copies in lung tissue were measured by quantitative PCR at 24 hours post-infection. (e) Neutrophils in BAL were enumerated by cytospin assay and neutrophil elastase protein was measured in BAL by ELISA at 8 hours post-infection. (f) MUC5AC protein in BAL was measured by ELISA at day 7 post-infection. Data represents mean (+/-SEM) of 5-8 mice per treatment group, representative of at least two independent experiments. Data were analysed by one-way ANOVA with Bonferroni post-test. ns = non-significant. *p<0.05, **p<0.01, ***p<0.001.

S

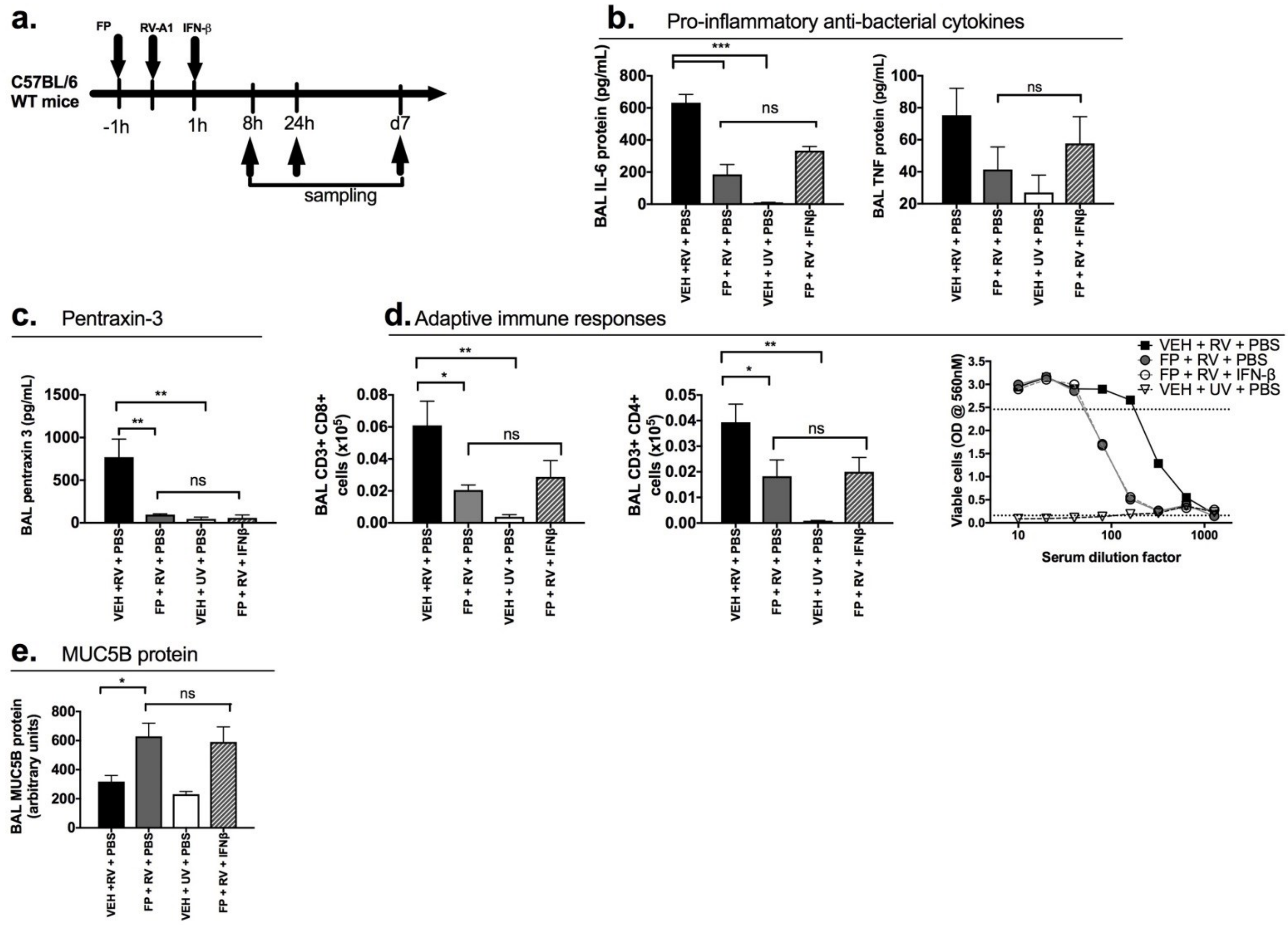

Supplementary figure 4: IFN-β does not reverse FP suppressed of other anti-bacterial responses nor restore acquired immunity or MUC5B. (a). C57BL/6 mice were treated intranasally with fluticasone propionate (20μg) or vehicle DMSO control and challenged intranasally with rhinovirus(RV)-A1B or UV-inactivated RV-A1B (UV). One hour after RV challenge, mice were additionally treated intranasally with 10^4 units recombinant IFN-β. (b) IL-6 and TNF proteins at 8 hours and (c) pentraxin-3 protein at 24 hours were measured in BAL by ELISA. (d) BAL cells were stained with antibodies specific for CD3, CD8 and CD4 and analysed by flow cytometry. BAL CD3+ CD8+ T cell numbers at day 2 post-infection and BAL CD3+ CD4+ T cell numbers at day 7 post-infection were enumerated. Sera were assayed for their ability to prevent cytopathic effect caused by the same RV serotype used for *in vivo* challenge. Cytopathic effect was quantified by crystal violet staining. Top dotted line: serum only, (uninfected) controls. Bottom dotted line: virus infected (no serum) control. (e) MUC5B protein was measured in BAL at day 7 post-infection by ELISA. In (a-c), (d, left 2 panels) and (e), data represents mean (+/-SEM) of 5-8 mice per treatment group, representative of at least two independent experiments. Data were analysed by one- or two-way ANOVA with Bonferroni post-test. ns = non-significant; *p<0.05; **p<0.01, ***p<0.001. In (d, right panel) data points represent sera pooled from 5 mice per group, representative of 2 independent experiments.

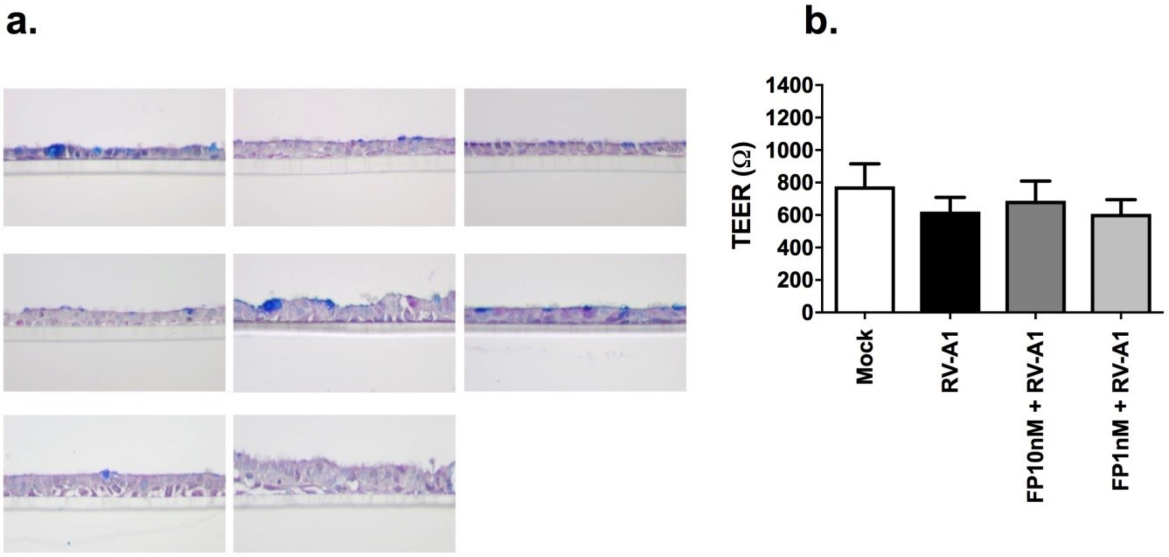

Supplementary figure 5: Histology and Trans-epithelial electrical resistance of bronchial epithelial cells from patients with COPD. (a) Airway epithelial cells from patients with COPD were fixed in 10% formalin, paraffin embedded, sectioned and stained using AB-PAS to confirm differentiation and presence of ciliated epithelial cells and mucus producing cell. Each panel represents a separate donor. Scale bars: 50μm, Magnification x 400. (b) Trans-epithelial electrical resistance (TEER) was measured using an epithelial voltohmmeter with AC current through an electrode set placed in the apical and basal media. An average of three readings was recorded at 96 hours post-infection. Mean (+/- SEM) shown for each treatment group.

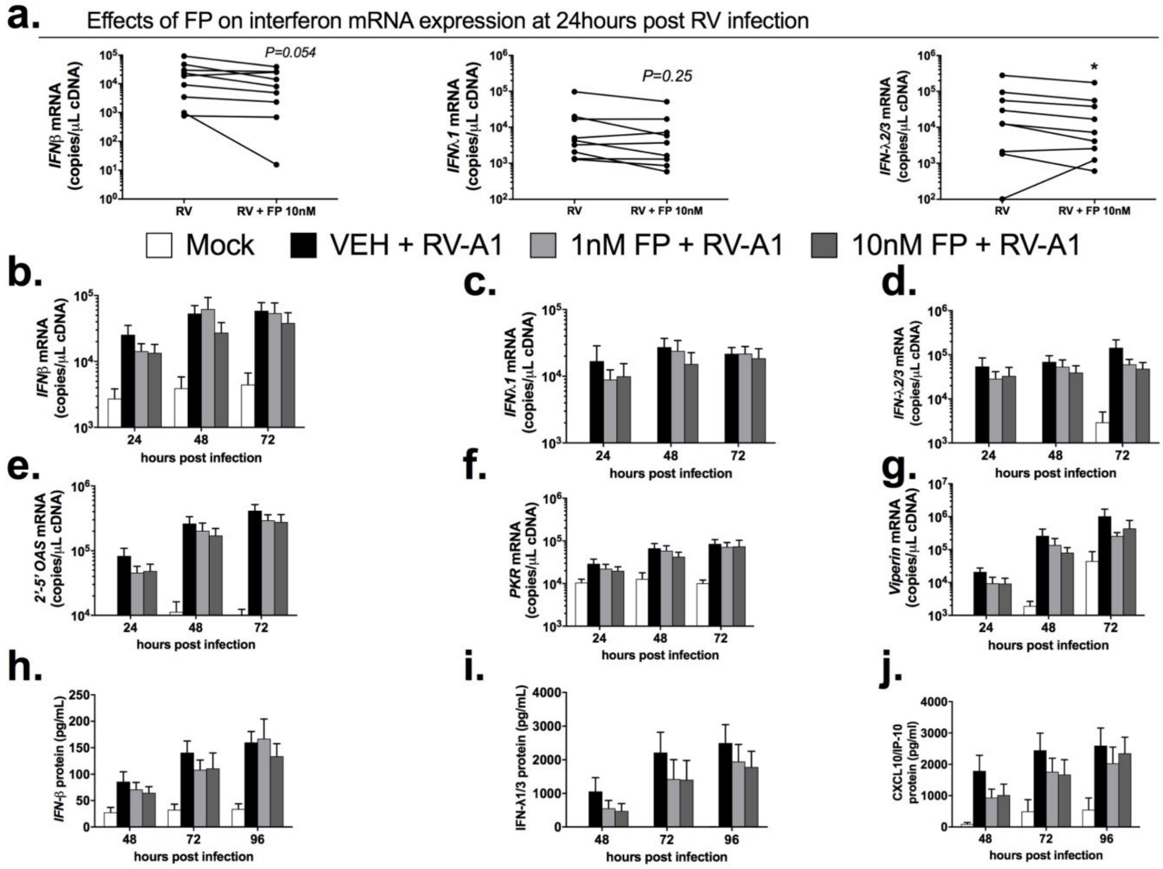

Supplementary Figure 6: Full dataset of experiments evaluting effect of FP on *ex vivo* RV infection in COPD cells

Primary airway epithelial cells (AECs) from patients with GOLD stage III COPD were cultured at the air-liquid interface and then treated *in vitro* with fluticasone propionate at 1nM and 10nM concentrations or vehicle control and infected with rhinovirus (RV)-A1. Cell lysates and supernatants were harvested at the indicated timepoints. (a) *IFNβ, IFNλ1* and *IFNλ2/3* mRNAs were measured at 24 hours post-infection. Full timecourse for (b) *IFNβ,* (c) *IFNλ1*, (d) *IFNλ2/3* mRNAs and ISGs (e)*2'-5' OAS,* (f) *PKR* and (g) *viperin* mRNAs in cell lysates measured by quantitative PCR. Full timecourse for (h) IFN-β, (i) IFN-λ1/3 and (j) CXCL10/IP-10 proteins in cell supernatants measured by ELISA. *$p<0.05$.

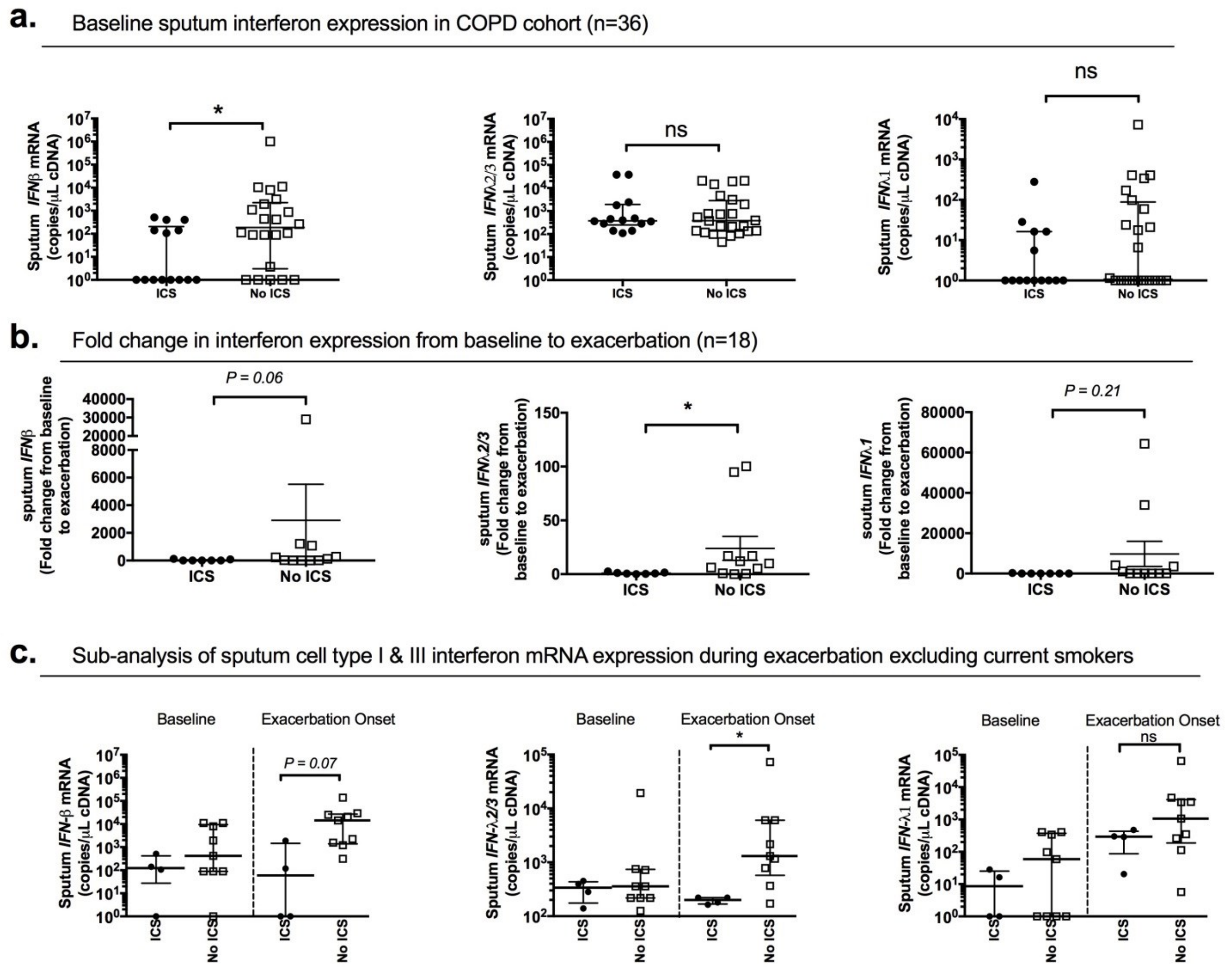

Supplementary figure 7: Baseline and fold change of sputum interferon expression and sub-analysis of patients who are not current smokers. Patients with COPD were monitored prospectively. Sputum samples were taken during stable state (baseline), at presentation with exacerbation associated with positive virus detection and 2 weeks during exacerbation. (a) Baseline *IFNβ*, *IFNλ1* and *IFNλ2/3* mRNA expression in sputum cells from the whole cohort (n=36) (b) Fold change from baseline to exacerbation for sputum cells for *IFNβ*, *IFNλ1* and *IFNλ2/3* mRNAs. (c) Sputum cell *IFNβ*, *IFNλ1* and *IFNλ2/3* mRNAs measured in a sub-analysis of patients with virus positive exacerbation with exclusion of current smokers. Data represents median (+/-IQR) per group. Data in (a-b) were analysed by Mann Witney U test. Data in (c) were analysed by Kruskall Wallis test with Dunn's post-test. ns = non-significant; *p<0.05.

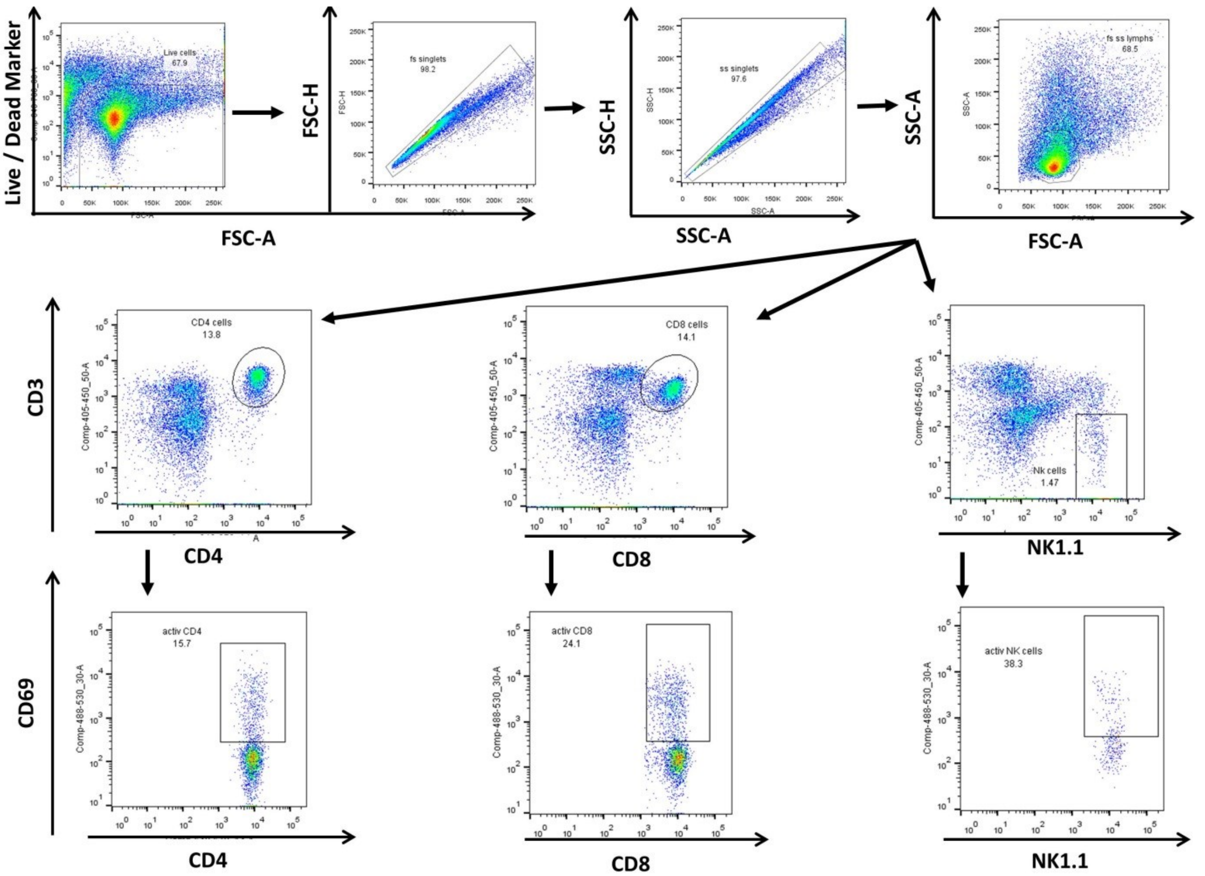

Supplementary Figure 8: Representative flow cytometric plots used for analysis of cell surface staining shown in Figure 1, 2 and Supplementary Figure 4. Abbreviations: FSC-A Forward scatter area; SSC-A Side scatter area; SSC-H Side scatter height

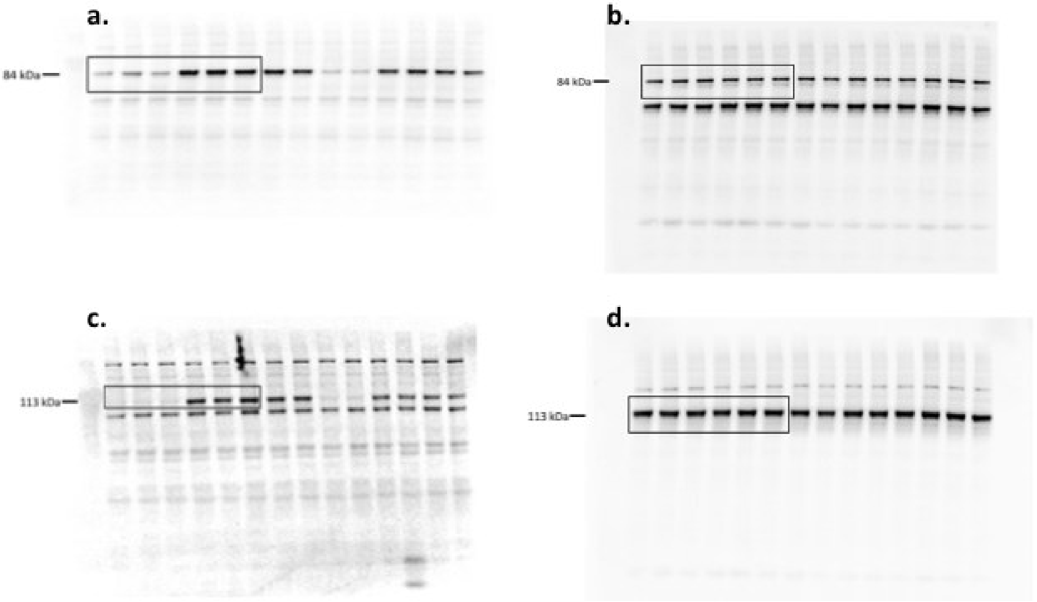

Supplementary Figure 9: Uncropped western blot images used in Fig. 3. BEAS-2B cells were treated with fluticasone propionate at 1nM and 10nM concentrations and stimulated with recombinant IFN-β. Cell extracts were harvested at 1 hour post-stimulation and prepared and analysed by Western blotting with antibodies to (a) pSTAT1 Y702 (b) STAT1 (c) pSTAT2 Y690 and (d) STAT2. Sections of blots used in Fig. 3 are highlighted.
